# Renalase deficiency suppresses hepatic triglyceride accumulation in the progression to MASLD/MASH by GAN diet in male mice

**DOI:** 10.14814/phy2.70720

**Published:** 2026-01-20

**Authors:** Yota Okano, Katsuyuki Tokinoya, Kai Aoki, Yuri Kato, Yasushi Kawakami, Kazuhiro Takekoshi

**Affiliations:** ^1^ Graduate School of Comprehensive Human Sciences University of Tsukuba Tsukuba Ibaraki Japan; ^2^ College of Education Yokohama National University Yokohama Kanagawa Japan; ^3^ Department of Health and Nutrition, Faculty of Health Science Niigata University of Health and Welfare Niigata Japan; ^4^ Research Fellowship for Young Scientists Japan Society for the Promotion of Science Chiyoda‐ku Tokyo Japan; ^5^ Faculty of Medicine University of Tsukuba Tsukuba Ibaraki Japan

**Keywords:** NAFLD, NASH, renalase, triglyceride

## Abstract

Metabolic dysfunction‐associated steatotic liver disease (MASLD) is a growing public health concern characterized by hepatic triglyceride (TG) accumulation, inflammation, and fibrosis. Renalase is known for its role in blood pressure regulation and catecholamine metabolism, but recent evidence suggests broader cytokine‐like functions. Moreover, its involvement in MASLD remains unclear. In this study, we examined the effects of renalase deficiency on hepatic lipid metabolism in a Gubra Amylin NASH (GAN) diet‐induced MASLD model using renalase knockout (KO) mice. Our results show that renalase KO mice exhibited reduced hepatic TG levels, accompanied by decreased gene and protein expression of Srebf1 involved in lipid synthesis, and lower gene expressions of antioxidant and fibrosis markers in KO‐GAN compared with wild type (WT)‐GAN. Additionally, in vitro experiments using AML12 cells with renalase knockdown confirmed reduced intracellular TG accumulation and lipid synthesis gene expression. Notably, the phosphorylation of Akt was significantly reduced in the liver of renalase‐KO mice, indicating that Akt signaling plays a critical role in the observed decrease in hepatic TG levels. These findings suggest that renalase regulates hepatic lipid metabolism through the Akt–Srebf1 pathway, and its deficiency attenuates TG accumulation, suggesting that renalase may modulate early hepatic lipid deposition that progresses toward MASLD.

## 1 INTRODUCTION

The global prevalence of obesity has significantly increased in recent years due to the westernization of diets and rapid changes in lifestyle (Swinburn et al., 2011). Obesity causes weight gain and also leads to complex metabolic disorders, such as insulin resistance, dyslipidemia, and chronic inflammation, all of which adversely affect the whole body (Rohm et al., 2022; Zhang et al., 2023). One of the major consequences of obesity is the accumulation of triglycerides (TG) in the liver, which plays a central role in the development of metabolic dysfunction‐associated steatotic liver disease (MASLD) (Rinella et al., 2023). MASLD is characterized by excessive fat accumulation in the hepatocytes even in the absence of alcohol consumption (Loomba et al., 2021). MASLD is regarded as steatotic liver disease (SLD), in which fat accumulates in the liver, and metabolic dysfunction‐associated steatohepatitis (MASH), in which inflammation and fibrosis develop in addition to fat accumulation depending on the progression of the disease. If the disease progresses from non‐alcoholic fatty liver disease to MASH, it may worsen to cirrhosis and liver cancer, which are serious clinical problems (Byrne & Targher, 2015; Estes et al., 2018). The mechanism of MASLD/MASH pathogenesis is explained by the “two‐hit theory,” in which excessive lipid accumulation (1st hit) is followed by secondary damage due to oxidative stress and inflammatory cytokines (2nd hit), which is thought to promote the development of MASH (Day & James, 1998). This concept has been further refined by the “multiple‐hit hypothesis,” which emphasizes that various parallel insults—such as insulin resistance, adipokine imbalance, gut microbiota alterations, and genetic or epigenetic factors—synergistically contribute to disease onset and progression, rather than occurring in a simple sequential manner (Buzzetti et al., 2016; Tilg & Moschen, 2010). Based on this multifactorial and complex pathogenesis, novel therapeutic molecules that target a wide range of pathological processes such as fat accumulation, inflammation, and fibrosis are needed for MASLD/MASH treatment (Friedman et al., 2018; Rong et al., 2023; Xu et al., 2022).

Renalase is a flavin adenine dinucleotide‐dependent soluble monoamine oxidase found in various organs such as the kidney, heart, and liver (Xu et al., 2005). Its primary function is to control blood pressure via the metabolism of circulating catecholamines (Wu et al., 2011; Xu et al., 2005). In addition, renalase in the liver has been reported to respond to oxidative stress (Aoki et al., 2021; Li et al., 2016). Previous studies have shown that renalase expression increased in a mouse model with hepatic ischemia–reperfusion (I/R) injury and the HepG2 treated with H_2_O_2_ (Li et al., 2016). Moreover, recent findings have demonstrated that renalase expression was upregulated in response to fasting‐induced oxidative stress in the small intestine, where it localized mainly to the crypt region and protected Caco‐2 cells from H₂O₂‐induced oxidative damage through NF‐κB p65–mediated signaling (Aoki et al., 2021). Additionally, previous studies have revealed that it is a bioactive substance that functions through the regulation of intracellular signals such as Akt, ERK, and p38 via the PMCA4b (plasma membrane Ca^2+^‐ATPase isoform 4b) receptor (Wang et al., 2014, 2015). Among these, the Akt pathway plays a crucial role in lipid metabolism by regulating de novo lipogenesis through SREBP‐1c activation, which induces the expression of fatty acid synthase (FAS) and acetyl‐CoA carboxylase (ACC) (Lawan & Bennett, 2017; Matsuda et al., 2013). Disruption of Akt signaling has been linked to hepatic lipid dysregulation and the progression of MASLD/MASH (Huang et al., 2018). In our previous study, we demonstrated that a choline‐deficient diet induced MASH in renalase‐knockout (KO) mice, accompanied by decreased αSMA expression and reduced phosphorylation of Akt in liver tissue (Tokinoya et al., 2021). However, the choline‐deficient model does not induce obesity, thereby limiting its applicability to MASLD/MASH pathogenesis. Given that renalase influences Akt signaling, its deficiency may alter hepatic triglyceride synthesis and lipid metabolism under a Gubra Amylin NASH (GAN)‐induced obesity. C57BL/6J mice fed the GAN diet for 28 weeks have been reported to develop severe steatosis, marked NASH, and increased fibrosis, establishing an early disease phenotype suitable for modeling NASH progression (Mathias et al., 2022).

Therefore, we hypothesize that renalase plays a critical role in the pathogenesis of MASLD/MASH by regulating triglyceride metabolism through Akt–SREBP‐1c signaling pathways. However, the effects of renalase deficiency on hepatic lipid accumulation under obesity‐induced metabolic stress remain unclear. In this study, we fed a GAN diet to KO mice to induce MASLD/MASH with obesity and examined whether renalase deficiency exacerbates disease progression by impairing Akt signaling and altering hepatic triglyceride metabolism.

## 2 MATERIALS AND METHODS

### 2.1 Animals and experimental design

Frozen sperm derived from Rnls^tm1a(EUCOMM)Wtsi^ mice with LoxP sequences inserted at both ends of exon3 were purchased from the International Mouse Phenotyping Consortium (California, USA). In vitro fertilization with egg from C57BL/6J mice was performed. Cre mRNA was inserted into the fertilized egg by electroporation, and Rnls^tm1b(EUCOMM)Wtsi^ mice deficient in the renalase gene were generated. Then, two backcrosses were performed with C57BL/6J mice. The Renalase Wild type (WT) and Knockout type (KO) mice used in the experiments were male mice produced by mating heterozygous mice. The genotypes of these mice were identified via polymerase chain reaction (PCR) using two primers for genomic DNA obtained from the ears via alkaline extraction. The housing environment was maintained at a room temperature of 23.5 ± 2.5°C, a humidity of 52.5 ± 12.5%, and a 12:12‐h light–dark cycle. All the mice were fed a standard diet (MF 12 mm; Oriental Yeast, Tokyo, Japan) and water ad libitum until the start of the experiment. Forty male mice at 6 weeks of age (WT, *n* = 20; KO, *n* = 20) were randomly divided into four groups (*n* = 10 per group): WT‐normal diet (ND; MF 12 mm), WT‐Gubra Amylin NASH (GAN) diet (D09100310, Research Diets, New Jersey, USA), KO‐ND, and KO‐GAN. The GAN diet formulation, which contains 4.49 kcal/g, 40% kcal from fat, 22% kcal from 10% fructose, and 2% cholesterol. This diet differs from conventional high‐fat diets by including high fructose and cholesterol content to promote hepatic steatosis and early NASH‐like pathology (Mathias et al., 2022). The various mice groups were housed for 30 weeks under the same environmental condition, and their diet was replenished every 3 days. Mice were fasted for 4 h before dissection and then euthanized via cervical dislocation after inhaling an isoflurane solution (isoflurane: propylene glycol = 3:7). Before euthanasia, blood was collected from the inferior vena cava while under anesthesia. Blood glucose levels were measured using a blood glucose meter for laboratory animals (ForaCare Japan, Tokyo, Japan). The serum was settled on ice for at least 30 min and centrifuged at 4°C, 3000 rpm for 15 min. After euthanasia, liver and epididymal white adipose tissue (eWAT) were collected. The wet weight of each tissue was measured, frozen in liquid nitrogen, and then stored at −80°C until analysis. Mice with liver tumors at dissection were excluded from the analysis (WT‐GAN group, *n* = 2).

### 2.2 Intraperitoneal glucose tolerance test (IPGTT)

IPGTT was conducted 1 week before dissection. Blood samples were collected by cutting off the tip of the tail at 1–2 mm, and the first drop of blood was removed to avoid contamination with tissue fluid. After baseline blood glucose levels were measured using a blood glucose meter for laboratory animals (ForaCare Japan, Tokyo, Japan), glucose (20% D(+)‐glucose, 1 mg/g BW) was injected intraperitoneally, and blood glucose levels were measured at 15, 30, 60, and 120 min.

### 2.3 Biochemical analysis

Serum aspartate transferase (AST), alanine aminotransferase (ALT), triglyceride (TG), total cholesterol (T‐CHO), and free fatty acid (NEFA) levels were measured using Oriental Yeast Co. Serum insulin levels were also measured using the Ultra‐sensitive “PLUS” Mouse Insulin Assay Kit (Cat#M1105; Takara, Shiga, Japan) following the manufacturer's instructions. The measured serum insulin concentrations were used, together with the blood glucose levels obtained at the time of dissection, to calculate the HOMA‐IR.

### 2.4 Thiobarbituric acid reactive substances (TBARS)

TBARS measurement in liver tissue was performed as described previously (Tokinoya et al., 2021). Briefly, NP‐40 buffer (1% NP‐40, 150 mM NaCl, 50 mM Tris–HCl pH 7.4, and 1 mM EDTA) containing Protease Inhibitor Cocktail Set III (Cat#163‐26061; Wako, Osaka, Japan) was added to the liver tissue and homogenized, and the homogenized supernatant was used as a sample. The absorbance at 532 nm was measured using a microplate reader (Varioskan LUX; Thermo Fisher Scientific, Massachusetts, USA). To determine the TBARS concentration in the sample, a calibration curve was constructed based on the absorbance of the standard solution. The TBARS concentration was compensated for by the protein concentration measured using a BCA Protein Assay Kit (Cat#T9300A; Takara, Shiga, Japan).

### 2.5 Histological assessments

The liver lobe was fixed with 4% paraformaldehyde phosphate buffer at 4°C for overnight and then replaced with 70% ethanol. The liver samples were cut into 3 μm thick sections. Hematoxylin and eosin (HE) and Sirius red staining were performed using an all‐in‐one fluorescence microscope (BZ‐X810, KEYENCE, Osaka, Japan) equipped with a plan fluorite 4× objective (NA0.13, 972081, KEYENCE, Osaka, Japan) and 20× objective (NA0.75, 971962, KEYENCE, Osaka, Japan). Images were analyzed by the BZ‐X Analyzer software (BZ‐H4A, KEYENCE, Osaka, Japan). In addition, steatosis, ballooning, inflammation, and fibrosis scores were evaluated by an independent examiner using a single‐blinded assessment based on previously established criteria (SAF score) (Pierre et al., 2012).

### 2.6 AML12 cell culture and in vitro cellular model

AML12 (alpha mouse liver 12), a normal mouse liver cell line, was purchased from the American Type Culture Collection (Cat#CRL‐2254, Lot#70051517; Carlsbad, CA, USA). AML12 cells were maintained in DMEM/Ham's F‐12 medium (Cat#048‐29785; Fujifilm, Tokyo, Japan) supplemented with 10% fetal bovine serum (FBS), 10 μg/mL of insulin, 5.5 μg/mL of transferrin, 5 ng/mL of selenium, 40 ng/mL of dexamethasone, and 1% penicillin/streptomycin solution at 5% CO_2_ and 37°C. Cells were seeded in 12 well plates (Cat#P12F01S; AS ONE, Osaka, Japan) at 4.0 × 10^5^/well and infected with adenovirus after reaching 90% confluence. An shRNA‐expressing adenovirus vector (AdV) was used for renalase knockdown (KD), and an AdV‐containing mCherry was used as a control vector. AML12 cells were infected with the adenovirus at a multiplicity of infection (MOI) of 10 for 6 h.

Oleic acid (Cat#O7501; Sigma‐Aldrich, Missouri, USA) and palmitic acid (Cat#P9767; Sigma‐Aldrich, Missouri, USA) were dissolved in ultrapure water (70°C, 5 min) and diluted to 1 mM (oleic: palmitic ratio 2:1) in serum‐free medium containing 1% fatty acid‐free bovine serum albumin (Cat#017‐15141; Fujifilm, Tokyo, Japan). After pre‐incubation in 1% serum medium for 6 h, 1 mM FFA was added for 24 h. Control cells were maintained in a 1% serum medium containing 1% fatty acid‐free bovine serum albumin. Oil Red O staining, triglyceride extraction, and RNA extraction were performed after treatment. All AML12 cell experiments were performed in technical triplicates from the same biological sample.

### 2.7 Preparation of adenovirus vectors

Adenovirus vector production plasmids with mCherry (VB010000‐0021kwc) or Rnls shRNA (VB900138‐9658acm) constructs were purchased from Vector Builder (Chicago, IL, USA). Each plasmid was inserted into *Escherichia coli* (VB UltraStableTM Chemically Competent Cells, VectorBuilder Chicago, USA) and cultured in LB medium. Each plasmid was extracted using NucleoBond Xtra Midi (Cat#740410; Takara, Shiga, Japan). Subsequently, each plasmid was digested with Pac I restriction enzyme (Cat#R0457L; New England Biolabs, Ipswich, USA) and the linearized plasmid was co‐transfected into HEK293A (Cat#R70507, Lot#2475537; Invitrogen, Waltham, USA) cells using PEI MAX™ (Cat# 24765; Polysciences, *Inc*., Warrington, USA), followed by incubation for several days. Viruses were collected from the supernatant of the culture medium via ultracentrifugation. The functional titer (MOI) of the collected viruses was determined using the Adeno‐X Rapid Titer Kit (Cat#632250; Takara, Shiga, Japan).

### 2.8 Oil red O staining

AML12 cells were washed with phosphate buffered saline (PBS) and fixed in 4% paraformaldehyde for 10 min. After washing with PBS, the cells were dehydrated with 60% isopropanol for 1 min. The cells were stained with 60% Oil Red O solution (Cat#1931; Sigma‐Aldrich, Missouri, USA) for 20 min. After washing with 60% isopropanol, the cells were washed twice with PBS. The cells were then performed in PBS using an all‐in‐one fluorescence microscope (BZ‐X710, KEYENCE, Osaka, Japan) with a plan fluorite 4× objective (NA0.13, 972081, KEYENCE, Osaka, Japan) and analyzed by the BZ‐X Analyzer software (BZ‐H4A, KEYENCE, Osaka, Japan). Afterward, the PBS was removed, and isopropanol was added to extract the Oil Red O. Oil Red O extracts were measured using a microplate reader (Varioskan LUX) at an absorbance of 492 nm.

### 2.9 Triglyceride measurement

Triglyceride (TG) in liver tissue and AML12 cells was extracted using the Folch method (Folch et al., 1957). TG was analyzed using LabAssay Triglyceride (Cat#291‐94501; Fujifilm, Tokyo, Japan). TG concentration was compensated for by the protein concentration measured using the BCA Protein Assay Kit (Cat#T9300A; Takara, Shiga, Japan).

### 2.10 Western blot analysis

Liver tissue was homogenized in NP‐40 buffer (1% NP‐40, 150 mM NaCl, 50 mM Tris–HCl pH 7.4, and 1 mM EDTA) containing Protease Inhibitor Cocktail Set III (Cat#163–‐26061; Fujifilm, Tokyo, Japan) and PhosSTOP (Cat#12352204; Roche, Basel, Switzerland). After homogenization, the samples were centrifuged at 4°C, 15,000*g*, and 15 min, and the supernatant was collected. The protein concentration of each extracted sample was measured using the BCA Protein Assay Kit (Cat#T9300A; Takara, Shiga, Japan) and a microplate reader (Varioskan LUX) at an absorbance of 562 nm. Each sample was diluted with loading buffer (4% SDS, 10% 2‐mercaptoethanol, 20% glycerol, 0.004% bromophenol blue, 0.125 M Tris HCl pH 6.8) to a concentration of 1 μg/μL and incubated at 95°C for 5 min. Ten microliters of each sample were loaded per lane, and electrophoresis was performed at 200 V for 30 min. After electrophoresis, the isolated proteins were transferred to a polyvinylidene fluoride (PVDF) membrane using a semidry blotting system (TransBlot® Turbo™ Transfer System Bio‐Rad, California, USA) at 1.3 A for 10 min. The PVDF membranes were blocked with Tris‐buffered saline [TBS‐T] containing 0.1% Tween‐20 containing 5% skim milk and 5% Blocking One (Nacalai, Kyoto, Japan) for 1 h at room temperature. After rinsing with TBS‐T several times, a primary antibody reaction was performed at 4°C overnight. The PVDF membranes were washed three times with TBS‐T for 10 min, followed by a secondary antibody incubation at room temperature for 1 h. The PVDF membranes were washed three times with TBS‐T for 10 min. Signals were detected using chemiluminescence reagents (Cat#2332638; EzWestLumi Plus; Atto, Tokyo, Japan) and FusionFX7.EDGE (Vilber Lourmat, Marne‐la‐Vallée, France). Table S1 shows the primary and secondary antibodies used in this study.

### 2.11 Quantitative real‐time PCR (qPCR)

To extract total RNA, the liver tissue and AML12 cells were homogenized in Sepasol‐RNA I Super G (Cat#09379‐55; Nacalai, Kyoto, Japan). Chloroform was added and mixed vigorously; subsequently, the samples were allowed to stay at room temperature for 3 min and then centrifuged at 4°C, 12,000*g*, for 15 min. The aqueous layer was collected, and isopropanol was added, mixed, and allowed to stand for 10 min. The supernatant was removed after centrifugation at 4°C, 12,000*g*, and 10 min. Afterward, 70% ethanol was added and centrifuged at 4°C, 12,000*g*, for 5 min to remove the supernatant. Finally, UltraPure Distilled Water (Cat#10977015; Thermo Fisher Scientific, Massachusetts, USA) was added and incubated at 65°C for 5 min. The total RNA concentration and purity were measured using a spectrophotometer (Nanodrop OneC; Thermo Fisher Scientific, Massachusetts, USA). Samples were diluted to 100 ng/μL, and ReverTra Ace (Cat#TRT‐101; TOYOBO, Osaka, Japan) was added. Reverse transcription (37°C, 15 min; 50°C, 5 min; 4°C ∞) was then performed via thermal cycler (TP530; Takara bio, Shiga, Japan). After reverse transcription, cDNA was diluted 10‐fold with UltraPure Distilled Water. TB Green Premix Ex II (Cat#RR820; Takara, Shiga, Japan), primers, and UltraPure Distilled Water were added to perform qPCR using QuantStudio 5 (Thermo Fisher Scientific, Massachusetts, USA). qPCR amplification was set up as follows: initial denaturation at 95°C for 30 s, followed by 40 cycles of denaturation at 95°C for 5 s, and annealing and extension at 60°C for 30 s. Rplp0 (ribosomal protein lateral stalk subunit P0) was used as the housekeeping gene, and the quantification cycle (Cq) value of the target gene was standardized to that of the housekeeping gene. The results were analyzed using the ΔΔCt method adapted from Livak & Schmittgen (2001). The relative expression levels of the target genes were calculated as the relative value compared with that of the WT‐ND group or the control group. The primer sequences used in this experiment are shown in Table S2.

### 2.12 Statistical analysis

Data are presented as mean ± standard deviation of the mean (SD). Statistical analysis was performed using GraphPad Prism 10 (GraphPad Software, California, USA) with a two‐way analysis of variance (Genotype, Diet in vivo; Rnls KD, FFA in vitro). The IPGTT results were analyzed using a three‐way ANOVA (Time, Genotype, Diet). If the interaction was significant, Tukey's post‐hoc test was performed. The Student's *t*‐test was used to compare the Rnls gene expression between the WT‐ND and WT‐GAN groups. Similarly, Rnls gene expressions were compared between Scrambled and Rnls shRNA. The significance level was set to *p* < 0.05.

## 3 RESULTS

### 3.1 MASLD model using GAN diet (Figure 1)

**FIGURE 1:**
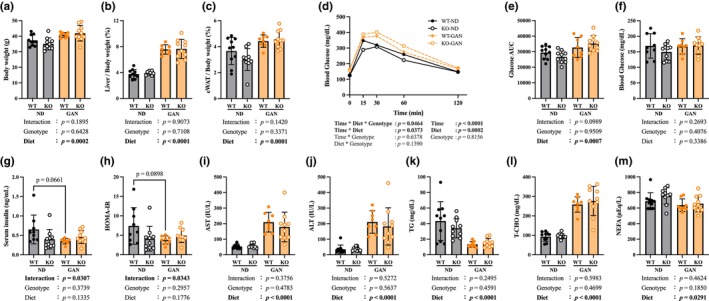
MASLD model by GAN loading. (a) Final body weight. (b) Ratio of liver weight to body weight. (c) Ratio of eWAT weight to body weight. (d) IPGTT, Intraperitoneal glucose tolerance test performed 1 week before dissection. (e) Area under the curve of IPGTT. (f) Blood glucose level for 4‐h fasting. (g) Serum insulin level for 4‐h fasting. (h) Homeostatic model assessment for insulin resistance (HOMA‐IR). (i) Serum AST, aspartate aminotransferase. (j) Serum ALT, alanine aminotransferase. (k) Serum TG, triglyceride. (l) Serum T‐CHO, total cholesterol. (m) Serum NEFA, nonesterified fatty acid. Data are shown as mean ± SD. *n* = 10 in WT‐ND, KO‐ND, and KO‐GAN group, *n* = 8 in WT‐GAN group.

After 30 weeks on a GAN diet, the GAN group showed significantly higher body weight than the ND group (Figure 1a). The liver weight and eWAT also increased significantly (Figure 1b,c). Results of the IPGTT performed 1 week before dissection showed that glucose intolerance was worse in the GAN group (Figure 1d,e). A three‐way ANOVA (Genotype × Diet × Time) revealed a significant interaction among the three factors, as well as a time × diet interaction and significant main effects of time and diet. Post hoc multiple comparisons (Tukey's test) indicated significant differences between diet groups at specific time points (see Supplementary File for full details). Fasting blood glucose levels showed no significant effects of genotype, diet, or their interaction (Figure 1f). In contrast, fasting serum insulin levels demonstrated a significant interaction between genotype and diet, although post hoc comparisons did not identify statistically significant differences between individual groups (Figure 1g). Consistent with this pattern, HOMA‐IR also exhibited a significant interaction, again without significant post hoc differences (Figure 1h). Serum AST, ALT, and T‐CHO levels, markers of liver function, were significantly increased in the GAN group (Figure 1i,j,l). In contrast, serum TG and NEFA levels were significantly decreased (Figure 1k,m).

### 3.2 Renalase deficiency suppresses lipid metabolism (Figure 2)

**FIGURE 2:**
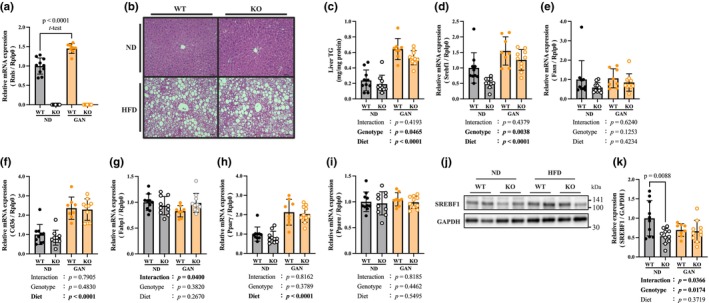
Renalase deficiency suppresses lipid metabolism. (a) Gene expression of renalase in each group. (b) HE, Hematoxylin and eosin staining of liver tissue. Scale bar: 100 μm at 10× magnification. (c) TG, Triglyceride concentration in liver tissue. (d) Gene expression of Srebf1, Sterol regulatory element binding transcription factor 1. (e) Gene expression of Fasn, Fatty acid synthase. (f) Gene expression of Cd36, cluster of differentiation 36. (g) Gene expression of Fabp1, Fatty acid binding protein 1. (h) Gene expression of Pparγ, Peroxisome proliferator‐activated receptor γ. (i) Gene expression of Pparα, Peroxisome proliferator‐activated receptor α. (j) Western blot image of SREBF1. (k) Protein expression of SREBF1. Data are shown as mean ± SD. *n* = 10 in WT‐ND, KO‐ND and KO‐GAN group, *n* = 8 in WT‐GAN group.

Renalase mRNA levels were determined using qPCR, and no gene expression was observed in the renalase KO group (Figure 2a). However, renalase mRNA levels were significantly higher in the WT‐GAN group than in the WT‐ND group (Figure 2a). Significant lipid droplets were observed in the HE staining of the liver tissue of the GAN group (Figure 2b). TG in the liver tissue was extracted and measured, and the results showed a significant increase in TG concentration in the GAN group compared to that in the ND group (Figure 2c). Furthermore, the TG concentration was significantly lower in the renalase KO group than in the WT group (Figure 2c). To evaluate lipid accumulation in the liver, we analyzed the expression of related genes involved in lipid synthesis (Srebf1, Fasn, Pparγ), lipid transport (Cd36, Fabp1), and lipid oxidation (Pparα) using qPCR (Figure 2d–i). mRNA levels of Srebf1, Cd36, and Pparγ significantly increased in the GAN group (Figure 2d,f,h). The mRNA level of Srebf1 significantly decreased in the renalase KO group compared to the WT group (Figure 2d). In addition, the protein expression of SREBF1 was also significantly lower in the KO‐ND group than in the WT‐ND group (Figure 2j,k).

### 3.3 Renalase deficiency partially decreased anti‐oxidant enzyme and suppresses intrahepatic inflammation (Figure 3)

**FIGURE 3:**
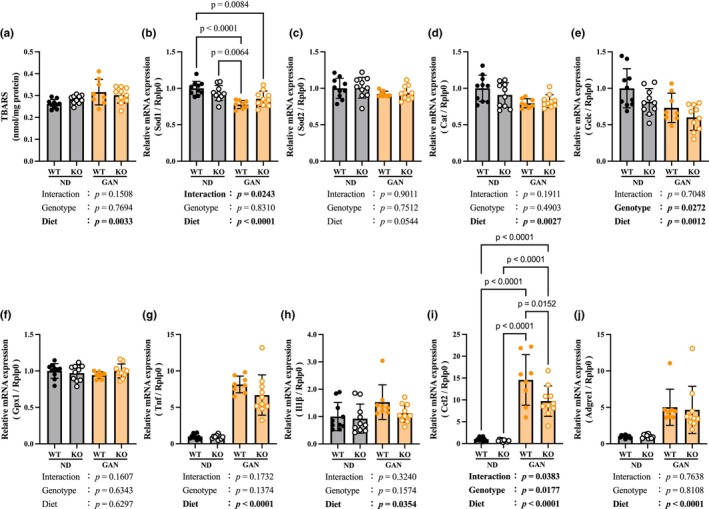
Increased oxidative stress due to GAN loading. (a) TBARS, Thiobarbituric acid reactive substances concentration in liver tissue. (b) Gene expression of Sod1, superoxide dismutase 1. (c) Gene expression of Sod2, superoxide dismutase 2. (d) Gene expression of Cat, Catalase. (e) Gene expression of Gclc, γ‐glutamylcysteine synthetase catalytic unit. (f) Gene expression of Gpx1, Gluthatione peroxidase 1. (g) Gene expression of Tnf, Tumor necrosis factor. (h) Gene expression of Il1β, Interleukin 1β. (i) Gene expression of Ccl2, C‐C motif chemokine ligand 2. (j) Gene expression of Adgre1, Adhesion G protein‐coupled receptor E1. Data are shown as mean ± SD. *n* = 10 in WT‐ND, KO‐ND and KO‐GAN group, *n* = 8 in WT‐GAN group.

We measured the TBARS concentration in the liver tissue to evaluate the oxidative stress caused by GAN. A significant increase in the TBARS concentration was observed in the GAN group, but no significant renalase deficiency effect was observed (Figure 3a). The expression levels of antioxidant‐related genes were also evaluated (Figure 3b–f). The mRNA levels of the antioxidant enzyme superoxide dismutase 1 (Sod1), which is found in the cytoplasm, and catalase (Cat) significantly decreased in the GAN group (Figure 3b,d). The mRNA level of the γ‐glutamylcysteine synthetase catalytic unit (Gclc), which is involved in the synthesis of the antioxidant glutathione, was similarly decreased (Figure 3e).

Significant increases in mRNA levels of inflammatory cytokines, Tnf, and Il1β were observed in the GAN group (Figure 3g,h). The mRNA levels of Ccl2, a chemokine, and Adgre1, a macrophage marker, also increased (Figure 3i,j). The mRNA expression of Ccl2 was significantly lower in the KO‐GAN group than in the WT‐GAN group (Figure 3i).

### 3.4 Renalase deficiency partially suppresses liver fibrosis (Figure 4, Table 1)

**FIGURE 4:**
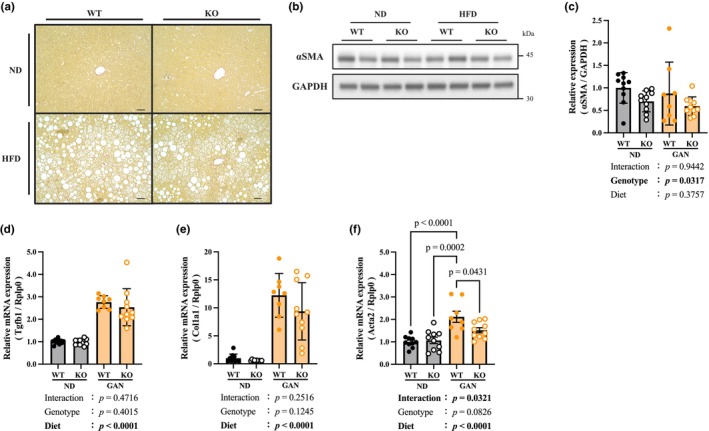
Renalase deficiency partially suppresses liver fibrosis. (a) Sirius Red staining of liver tissue. Scale bar: 100 μm at 10× magnification. (b) Western blot image of αSMA, α smooth muscle actin 2. (c) Protein expression of αSMA. (d) Gene expression of Tgfb1, Transforming growth factor‐β1. (e) Gene expression of Col1a1, Collagen type I alpha 1 chain. (f) Gene expression of Acta2. Data are shown as mean ± SD. *n* = 10 in WT‐ND, KO‐ND and KO‐GAN group, *n* = 8 in WT‐GAN group.

**TABLE 1:** SAF score.

Diet	Genotype	*n*	Steatosis score				Ballooning score			Inflammation score			Fibrosis score				
			0	1	2	3	0	1	2	0	1	2	0	1	2	3	4
ND	WT	10	10	‐	‐	‐	9	1	‐	4	6	‐	10	‐	‐	‐	‐
	KO	10	10	‐	‐	‐	10	‐	‐	5	5	‐	10	‐	‐	‐	‐
GAN	WT	8	‐	5	3	‐	3	5	0	1	6	1	4	4	‐	‐	‐
	KO	10	‐	2	8	‐	5	4	1	1	7	2	5	5	‐	‐	‐

No obvious fibrosis was observed in the GAN group based on Sirius Red staining of the liver tissue (Figure 4a). The protein expression of αSMA and mRNA levels of Acta2, a marker of hepatic stellate cell (HSC) activation, were significantly lower in the renalase‐KO group than in the WT group (Figure 4b,c,f). In contrast, the expression of liver fibrosis‐related genes (Tgfb1, Col1a1) was significantly increased in the GAN group (Figure 4d,e). Although the SAF score indicated clear progression of steatosis and inflammation, little evidence of fibrosis development was observed (Table 1).

### 3.5 Renalase deficiency suppresses intracellular signaling (Figure 5)

**FIGURE 5:**
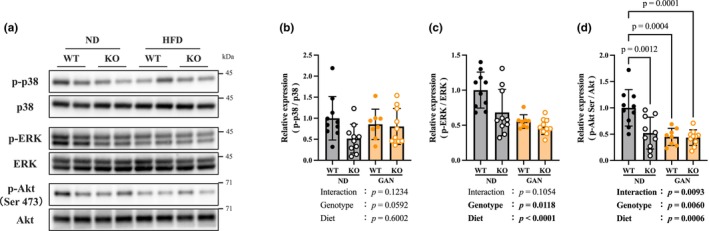
Renalase deficiency suppresses intracellular signaling. (a) Western blot images of p38, ERK, and Akt. (b) Phosphorylation level of p38. (c) Phosphorylation level of ERK, extracellular signal‐regulated kinase. (d) Phosphorylation level of Akt Ser473. Data are shown as mean ± SD. *n* = 10 in WT‐ND, KO‐ND, and KO‐GAN group, *n* = 8 in WT‐GAN group.

No significant differences in phosphorylated p38 level were observed between the GAN and renalase‐deficient groups (Figure 5a,b). Phosphorylated ERK and Akt levels significantly decreased in the GAN and renalase KO groups (Figure 5a,c,d). In particular, the phosphorylated Akt level was significantly lower in the KO‐ND group compared to the WT‐ND group (Figure 5d).

### 3.6 Renalase KD suppresses lipid metabolism in AML12 cells (Figure 6)

**FIGURE 6:**
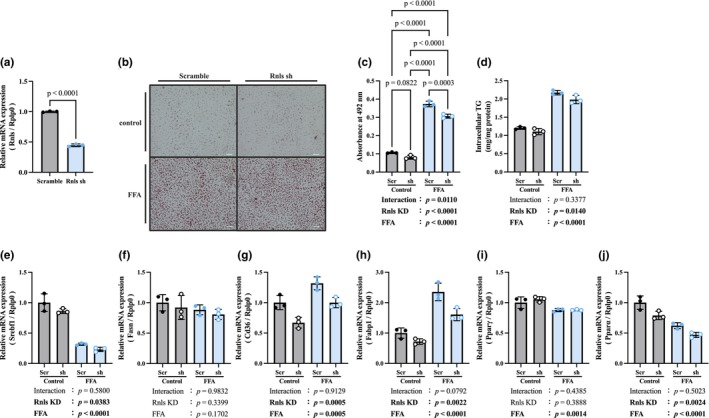
Renalase KD suppresses lipid metabolism in AML12 cells. (a) Efficiency of renalase knockdown. (b) ORO staining of AML12 cells. Scale bar: 50 μm at 20× magnification. (c) Quantification of ORO. (d) TG, triglyceride concentration in AML12 cells. (e) Gene expression of Srebf1, sterol regulatory element binding transcription factor 1. (f) Gene expression of Fasn, fatty acid synthase. (g) Gene expression of Cd36, cluster of differentiation 36. (h) Gene expression of Fabp1, fatty acid binding protein 1. (i) Gene expression of Pparγ, peroxisome proliferator‐activated receptor γ. (j) Gene expression of Pparα, peroxisome proliferator‐activated receptor α. Data are shown as mean ± SD. *n* = 3 in each group. Scr, scramble; sh, Rnls sh.

Renalase expression was knocked down by infection with an shRNA‐expressing adenovirus. In this study, renalase knockdown efficiency was approximately 50% (Figure 6a). ORO staining revealed that FFA treatment resulted in the accumulation of lipids (Figure 6b). ORO extracts were significantly decreased in the Rnls sh‐FFA group compared to those in the Scrambled‐FFA group (Figure 6c). The TG level in AML12 cells was a significant decrease was observed in the Rnls shRNA group compared to that in the Scramble group (Figure 6d). Furthermore, the mRNA levels of Srebf1, Cd36, Fabp1, and Pparα were significantly decreased by renalase KD and/or FFA treatment (Figure 6e,g,h,j). Additionally, the mRNA level of Pparγ was significantly decreased by FFA (Figure 6i). There was no significant difference between the two groups in the mRNA level of Fasn (Figure 6f).

## 4 DISCUSSION

In this study, we aimed to investigate the role of renalase in hepatic lipid metabolism under diet‐induced obesity. To model MASLD, we used the Gubra Amylin NASH (GAN) diet, which replicates key human features such as hepatic steatosis, metabolic abnormalities, and early fibrosis. GAN‐fed mice developed characteristic MASLD phenotypes, including hepatomegaly, adiposity, hyperlipidemia, glucose intolerance, and hepatic lipid accumulation, confirming successful model establishment (Boland et al., 2019; Hansen et al., 2020; Mathias et al., 2022), but did not exhibit a severity sufficient to progress to MASLD or fibrosis resulting from SAF score. Despite these systemic changes, renalase deficiency did not significantly affect body weight, liver mass, or metabolic parameters, suggesting a more specific role for renalase in hepatic lipid regulation. However, 4‐h fasting serum insulin levels showed a genotype‐dependent pattern: WT mice exhibited a tendency toward reduced insulin concentrations when fed the GAN diet compared with the ND diet, whereas KO mice maintained low insulin levels under both dietary conditions and were not affected by GAN feeding. This pattern contrasts with a previous report in which renalase inhibition restored insulin levels through improved β‐cell function (MacDonald et al., 2025). The previous studies were conducted in the context of genetic models or type 1 diabetes and did not examine the effects of diets used to induce MASLD. Therefore, future work is needed to evaluate the consequences of long‐term high‐fat diet feeding, which is more relevant to type 2 diabetes, as well as to investigate models in which renalase is not genetically deleted from birth—such as those employing pharmacological inhibition or inducible Cre recombinase–mediated knockout systems.

In MASLD, excessive uptake of dietary and adipose tissue‐derived free fatty acids (FFA) into the liver and increased de novo lipid biosynthesis lead to fat accumulation in hepatocytes (Ipsen et al., 2018). In the present study, we observed a decrease in TG concentration in the liver tissue and protein expression of Srebf1, a regulator of lipid synthesis, in renalase KO mice. In vitro experiments using adenovirus vector‐induced renalase knockdown (KD) AML12 cells showed that ORO quantification and intracellular TG levels were decreased by Rnls KD. The gene expression of Srebf1 decreased in a manner that is similar to that in the liver tissue. In addition, the expression levels of Cd36 and Fabp1, which are involved in fatty acid uptake, decreased. These results may reflect decreased fatty acid demand or a compensatory downregulation response to impaired lipogenesis, with the reduction in renalase expression itself further contributing to these alterations in lipid metabolic regulation. Therefore, renalase may be involved in lipid synthesis and transport pathways. These changes were most likely due to the reduced phosphorylation of Akt caused by renalase deficiency. Akt plays an important role in the transcription and activation of Srebf1 (Krycer et al., 2010). Our result showed that renalase deficiency significantly decreased phosphorylation of Akt in the ND group compared with WT mice. Akt activation via the PI3K pathway promotes Golgi migration of Srebf1 and the transcriptional activation of downstream genes involved in lipid synthesis (Yellaturu et al., 2009). Akt also improves Srebf1 stability by inactivating GSK3 and preventing the degradation of active Srebf (Bengoechea‐Alonso & Ericsson, 2009; Sundqvist et al., 2005). The decreased Akt phosphorylation and Srebf1 expression observed in the renalase‐KO mice in this study are consistent with these mechanisms. Therefore, renalase deficiency suppresses lipid metabolism via the Akt–Srebf1 pathway and suggests a potential role in attenuating the hepatic lipid accumulation that precedes MASLD pathogenesis.

In contrast to its effects on lipid metabolism, renalase deficiency had minimal effects on oxidative stress and inflammation, which were primarily elevated due to GAN exposure (Figure 3). However, renalase‐KO mice exhibited reduced expression of αSMA—a key marker of hepatic stellate cell activation and fibrosis—at both the mRNA and protein levels (Figure 4b,c,f). This finding is consistent with our previous findings using a choline‐deficient, L‐amino acid‐defined high‐fat diet (CDAHFD) model (Tokinoya et al., 2021). This observed reduction in αSMA was likely associated with suppressed ERK signaling (Figure 5a,c), in line with previous reports that inhibition of ERK signaling attenuates hepatic fibrosis (Jeng et al., 2020). These findings suggest that, in addition to its role in hepatic lipid regulation, renalase may contribute to fibrosis progression through ERK‐mediated stellate cell activation under metabolic stress.

This study has several limitations. First, although the GAN diet was used to induce MASLD, the phenotype did not fully reproduce the levels of steatosis, inflammation, or fibrosis reported in previous studies. Notably, compared with CDAHFD diets model (Tokinoya et al., 2021), the GAN diet int. this study produces a substantially milder hepatic injury profile. Our results exhibited less severe hepatic damage, which may explain the moderate pathological changes observed. However, this milder phenotype also represents a strength, as the long‐term GAN feeding used here generated a metabolic state that more closely resembles early‐stage human MASLD rather than the acute and severe injury seen in choline‐deficient models. It has also been reported that the GAN diet is highly suitable for characterizing the efficacy of novel NASH therapeutics in mouse models and for advancing preclinical drug development (Hansen et al., 2020). Despite these differences, hepatic lipid accumulation was evident, and the expression pattern of fibrosis‐related genes such as Acta2 remained directionally consistent with earlier findings, supporting a link between renalase deficiency and hepatic lipid and fibrotic regulation. Second, the AML12 cell experiments were based on technical replicates from an immortalized cell line, with identical passage numbers seeded into separate wells. As biological replication was not achieved, future validation using primary hepatocytes or human‐derived cells will be important to strengthen the mechanistic interpretation.

In conclusion, our findings demonstrate that renalase facilitates hepatic lipid accumulation through the Akt–Srebf1 signaling pathway. Its deficiency suppresses lipogenesis and fatty acid uptake, suggesting a potential protective role in preventing progression toward MASLD under dietary obesity. Additionally, these results position renalase as a metabolic regulator and a potential therapeutic target in MASLD. Further studies are needed to define the upstream modulators of renalase and explore the translational relevance of this pathway.

## AUTHOR CONTRIBUTIONS

Katsuyuki Tokinoya, Yasushi Kawakami, and Kazuhiro Takekoshi conceived and designed the research. Yota Okano, Katsuyuki Tokinoya, Kai Aoki, and Yuri Kato performed experiments. Yota Okano, Katsuyuki Tokinoya, Kai Aoki, and Yuri Kato analyzed data. Yota Okano, Katsuyuki Tokinoya, Kai Aoki, and Kazuhiro Takekoshi interpreted the results of experiments. Yota Okano, Katsuyuki Tokinoya, and Yuri Kato prepared figures. Yota Okano, Katsuyuki Tokinoya, Kai Aoki, Yuri Kato, Yasushi Kawakami, and Kazuhiro Takekoshi drafted the manuscript. All approved the final version of the manuscript.

## CONFLICT OF INTEREST STATEMENT

The authors declare that they have no conflict of interest.

## ETHICS STATEMENT

This study was approved by the Animal Care and Use Committee of University of Tsukuba (approval number: 24–161). The study was conducted following the ethical principles outlined in the Guide for the Care and Use of Laboratory Animals.

## Supporting information


**Table S1.** Lists of antibodies used in western blot analysis.

**Table S2.** Primer sequences used in qPCR.

Data S1.

## Data Availability

The datasets generated during and/or analyzed during the current study are available from the corresponding author upon reasonable request.
