# Supplementary material for: Renalase deficiency suppresses hepatic triglyceride accumulation in the progression to MASLD/MASH by GAN diet in male mice
Source: Physiol Rep. 2026 Jan 20;14(2):e70720. doi: 10.14814/phy2.70720 (PMC12819575; doi:10.14814/phy2.70720)
Supplement: Supplementary file 2 — Table S2. Primer sequences used in qPCR. [file PHY2-14-e70720-s001.docx]

Table S2. Primer sequences used in qPCR.
