# Supplementary material for: Renalase deficiency suppresses hepatic triglyceride accumulation in the progression to MASLD/MASH by GAN diet in male mice
Source: Physiol Rep. 2026 Jan 20;14(2):e70720. doi: 10.14814/phy2.70720 (PMC12819575; doi:10.14814/phy2.70720)
Supplement: Supplementary file 3 — Data S1. [file PHY2-14-e70720-s003.zip › PHYSREP-2025-07-681-s04.pdf]

SREBF1

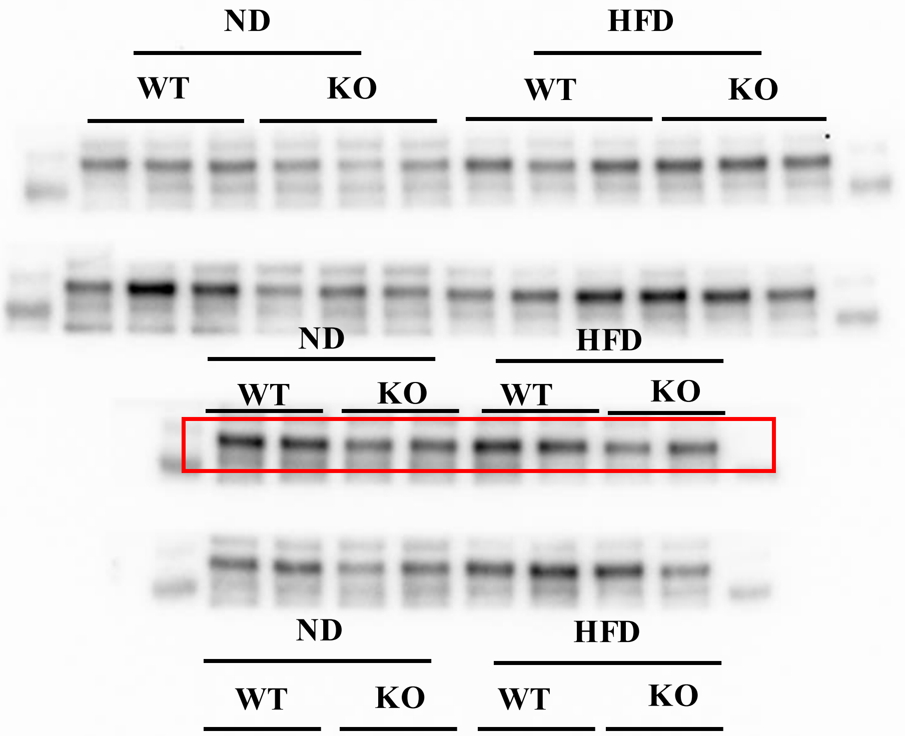
ND
HFD
WT
KO
WT
KO
ND
HFD
WT
KO
WT
KO
ND
HFD
WT
KO
WT
KO

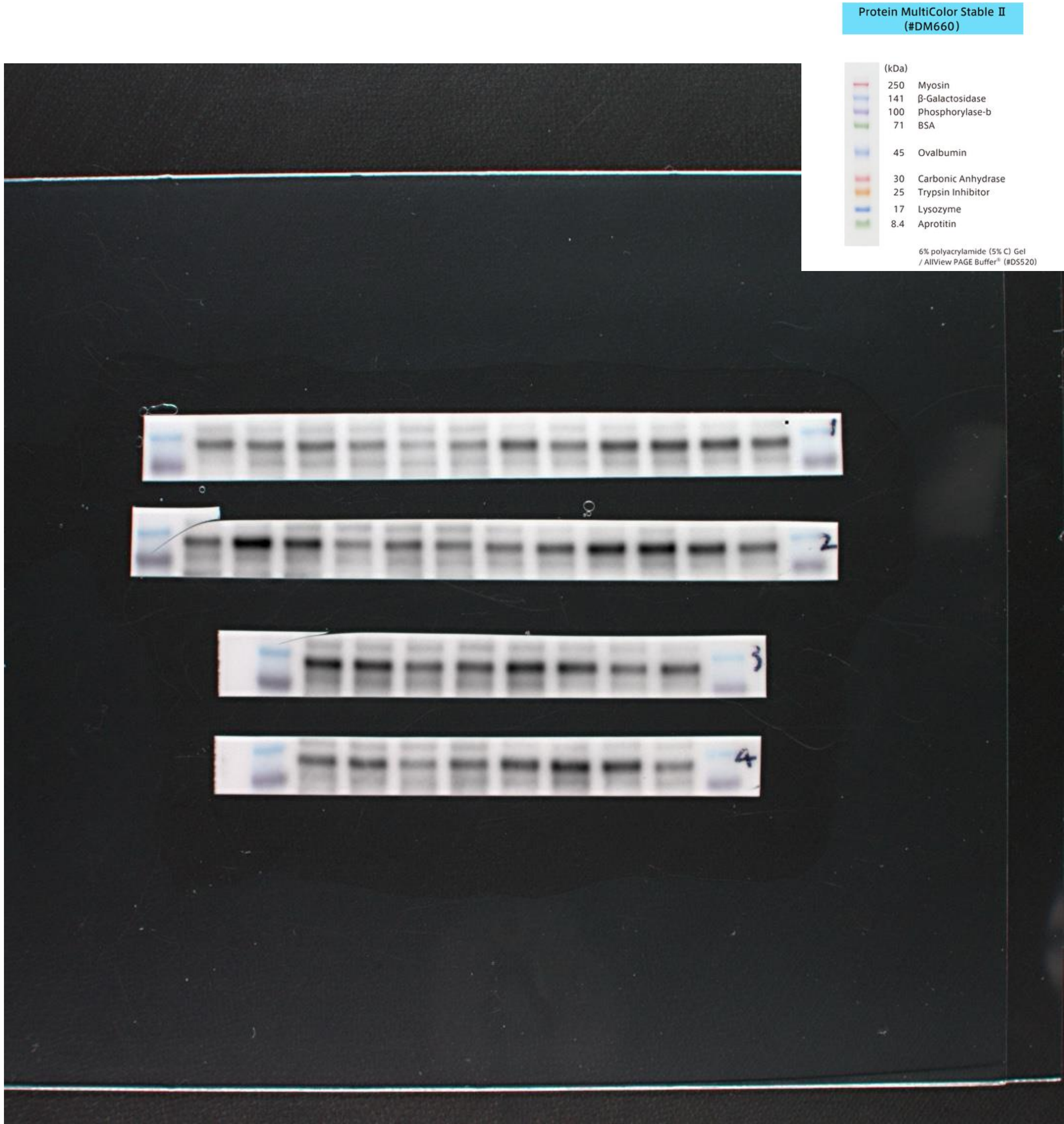
Protein MultiColor Stable Ⅱ (#DM660)
(kDa)
250 Myosin
141 β-Galactosidase
100 Phosphorylase-b
71 BSA
45 Ovalbumin
30 Carbonic Anhydrase
25 Trypsin Inhibitor
17 Lysozyme
8.4 Aprotitin
6% polyacrylamide (5% C) Gel / AllView PAGE Buffer® (#DS520)
1
2
3
4

GAPDH(SREBF1)

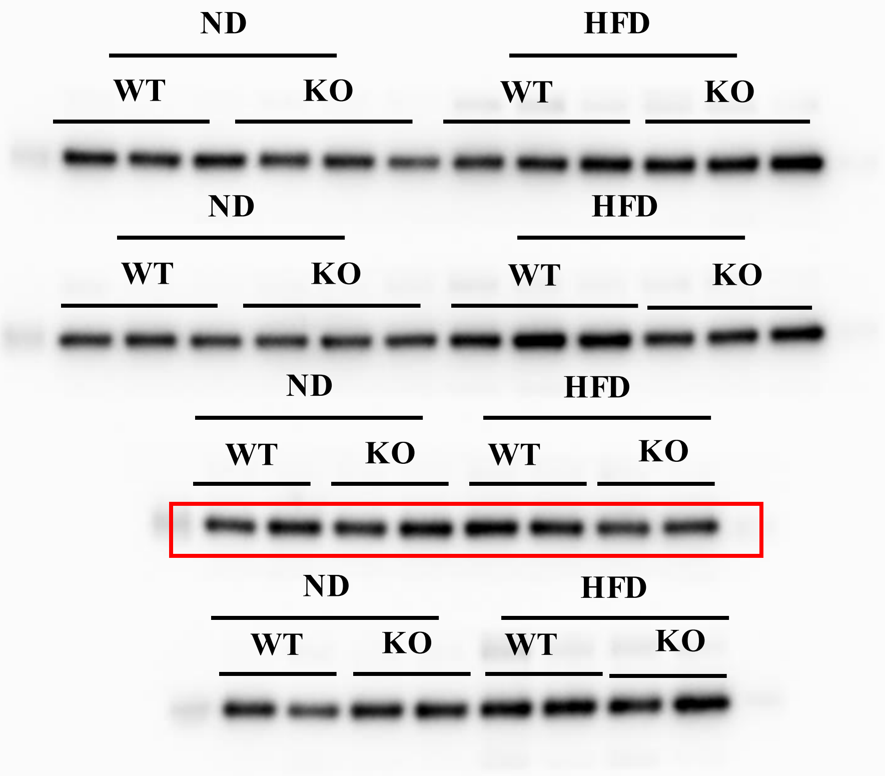

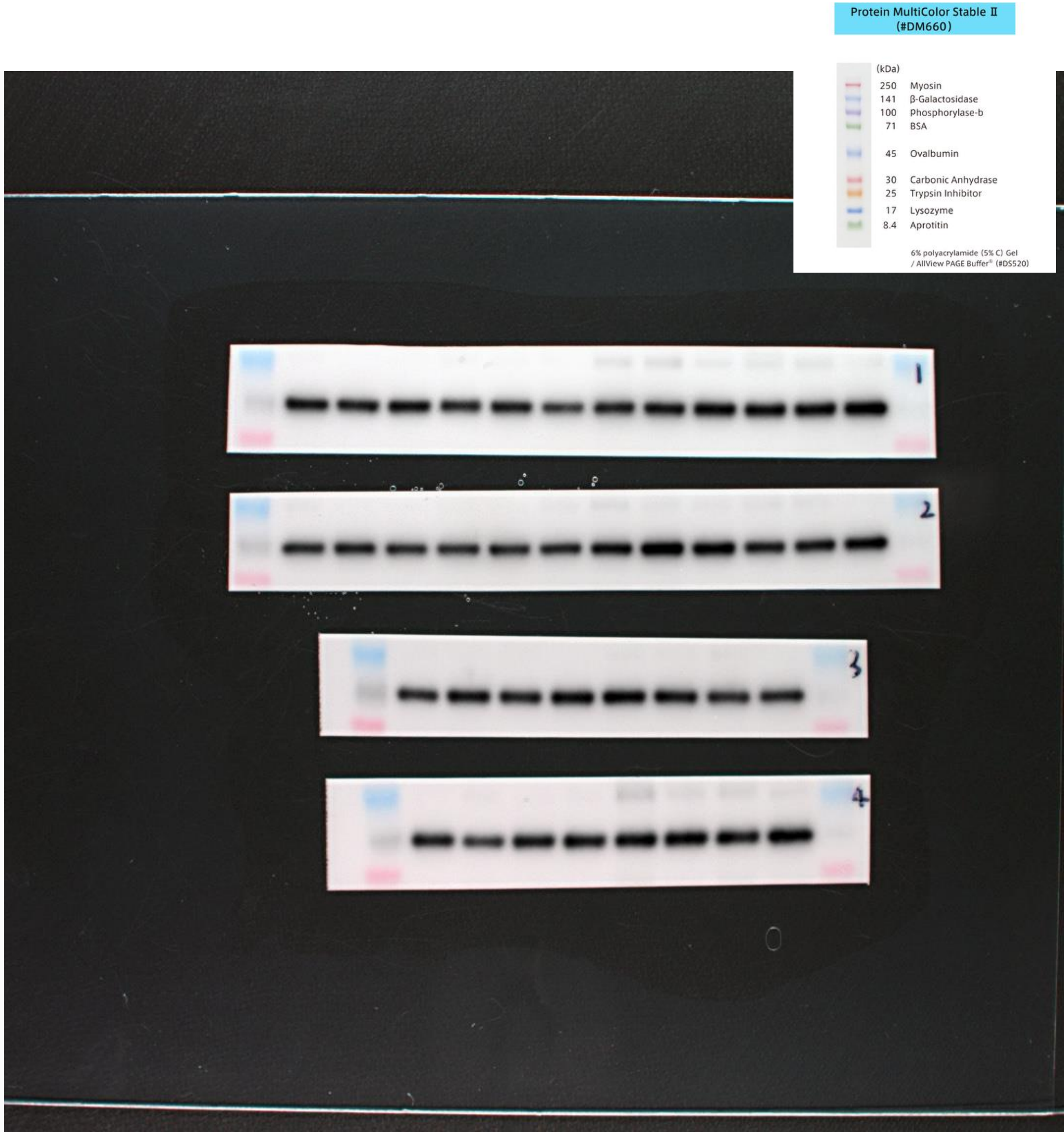

αSMA

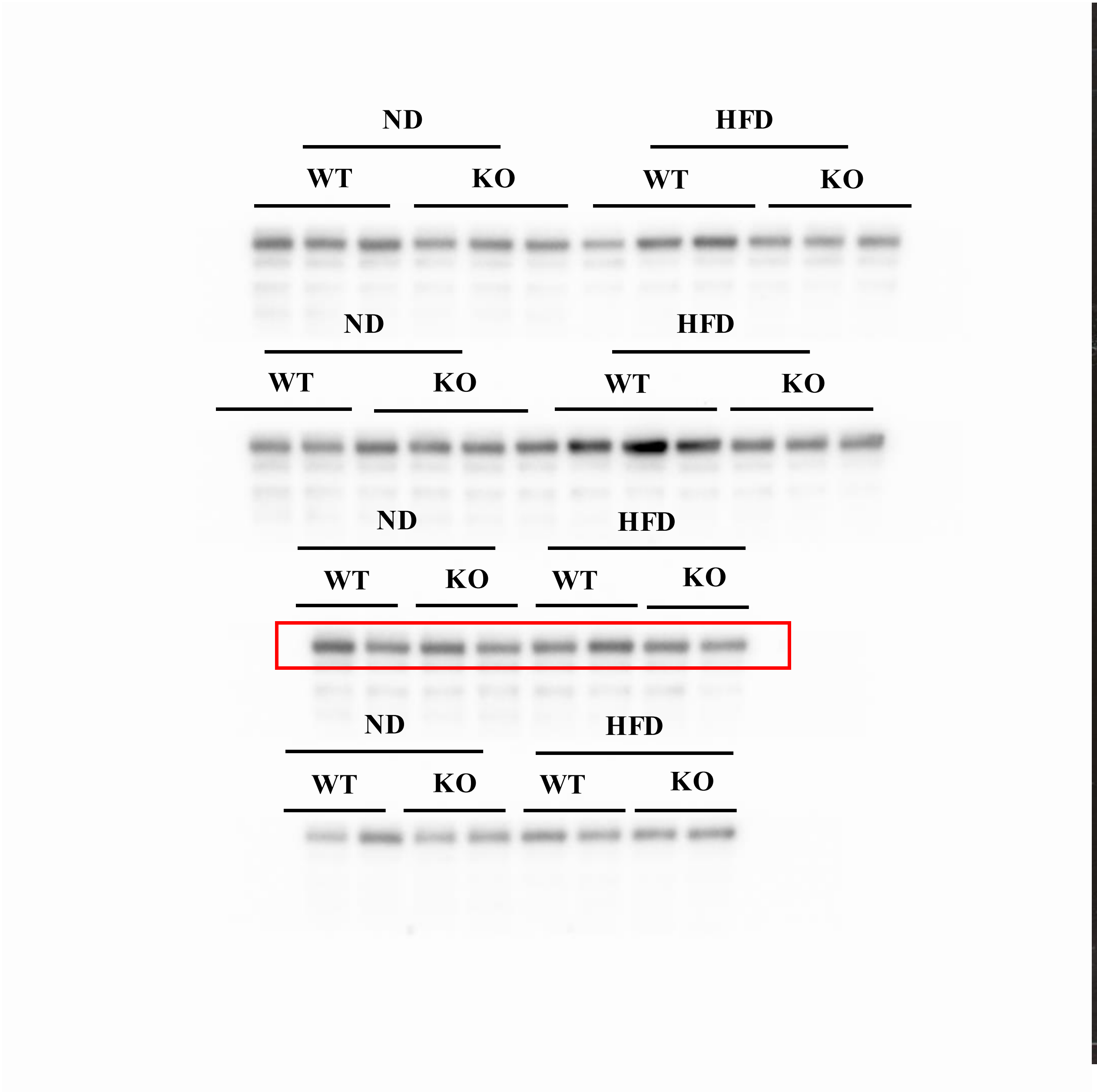

ND
HFD
WT
KO
WT
KO
ND
HFD
WT
KO
WT
KO
ND
HFD
WT
KO
WT
KO
ND
HFD
WT
KO
WT
KO

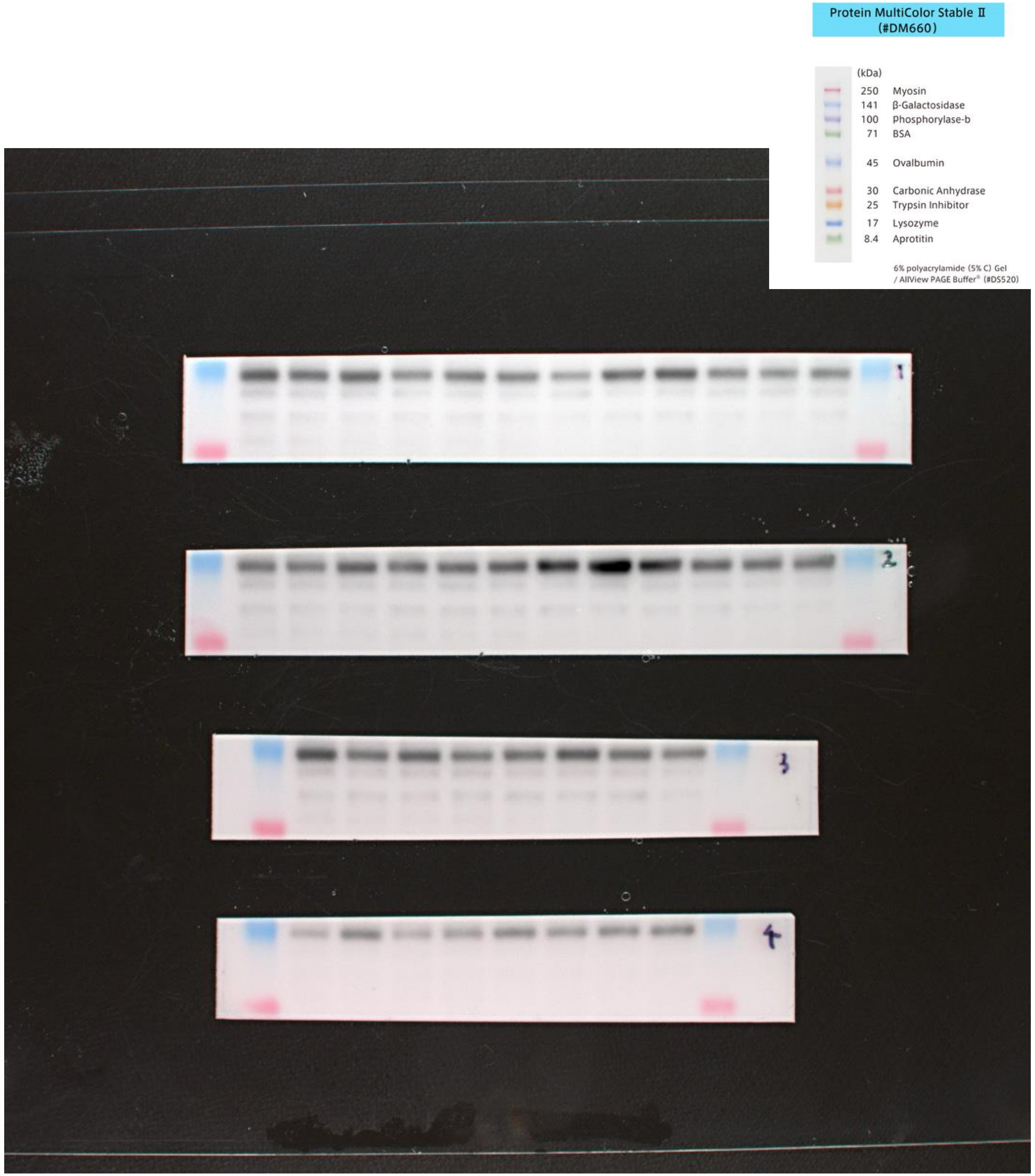

Protein MultiColor Stable Ⅱ (#DM660)
(kDa)
250 Myosin
141 β-Galactosidase
100 Phosphorylase-b
71 BSA
45 Ovalbumin
30 Carbonic Anhydrase
25 Trypsin Inhibitor
17 Lysozyme
8.4 Aprotitin
6% polyacrylamide (5% C) Gel / AllView PAGE Buffer® (#DS520)
1
2
3
4

GAPDH(αSMA)

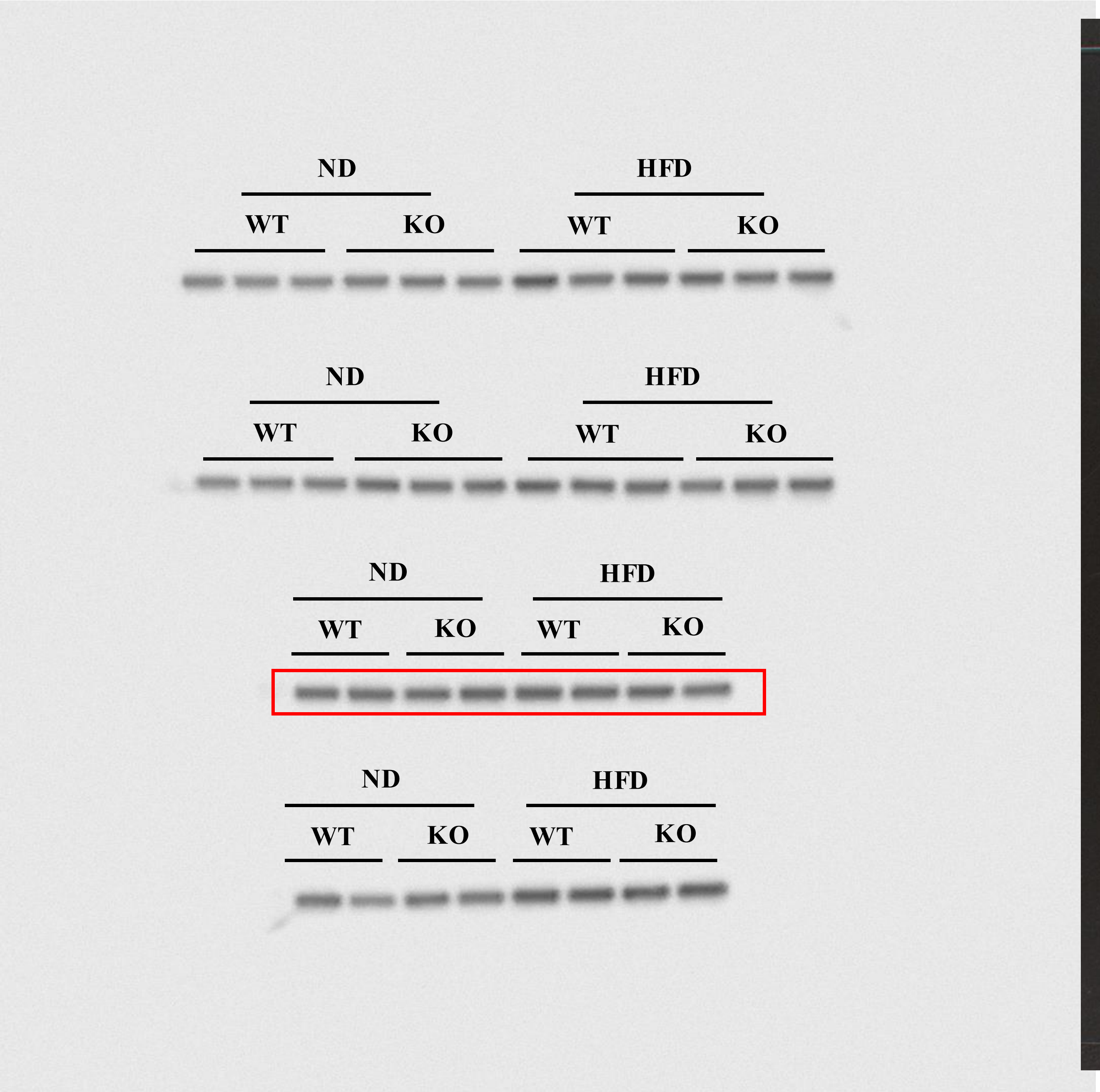
ND
HFD
WT
KO
WT
KO
ND
HFD
WT
KO
WT
KO
ND
HFD
WT
KO
WT
KO
ND
HFD
WT
KO
WT
KO

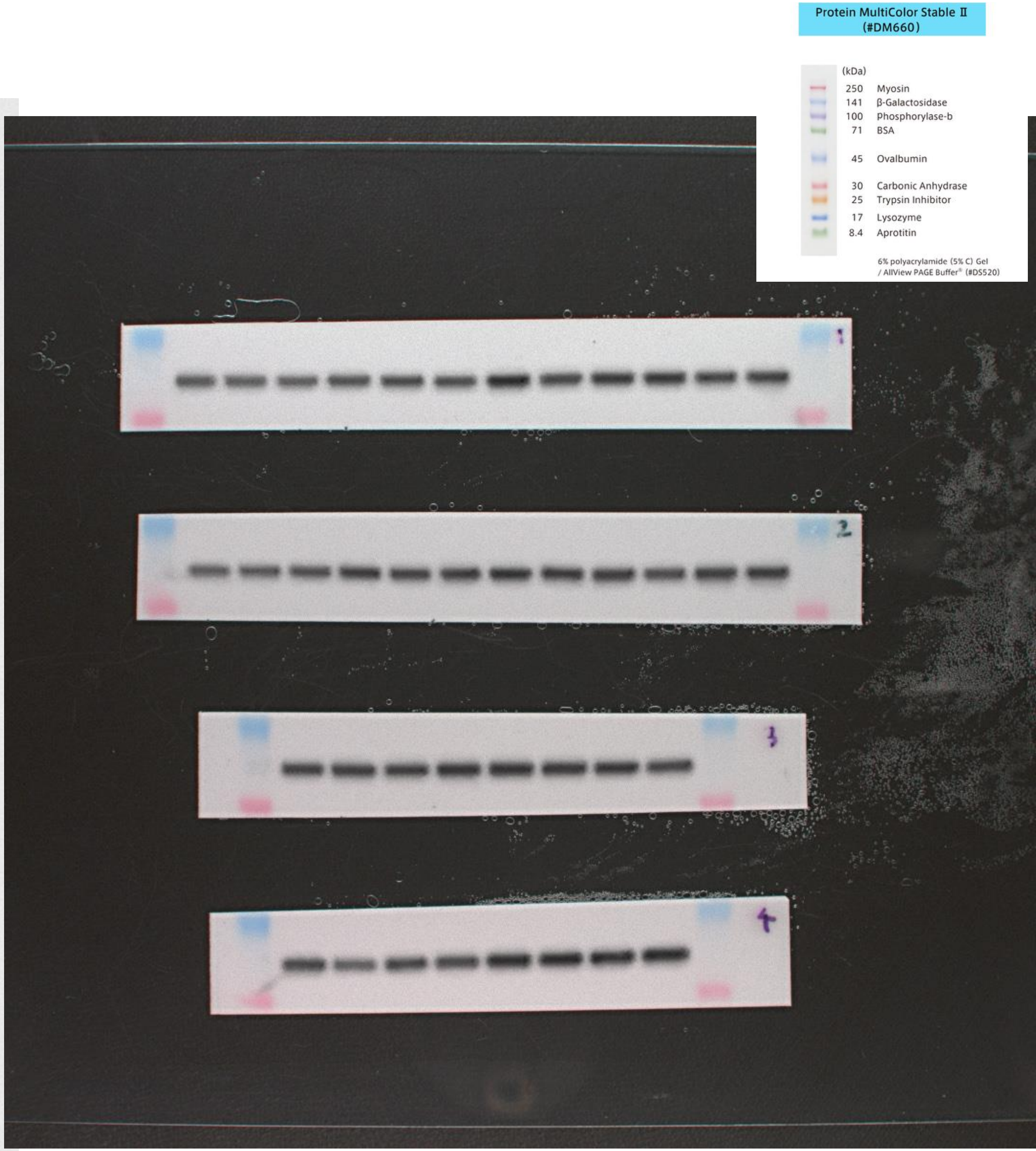
Protein MultiColor Stable Ⅱ (#DM660)
(kDa)
250 Myosin
141 β-Galactosidase
100 Phosphorylase-b
71 BSA
45 Ovalbumin
30 Carbonic Anhydrase
25 Trypsin Inhibitor
17 Lysozyme
8.4 Aprotitin
6% polyacrylamide (5% C) Gel / AllView PAGE Buffer® (#DS520)

p-p38

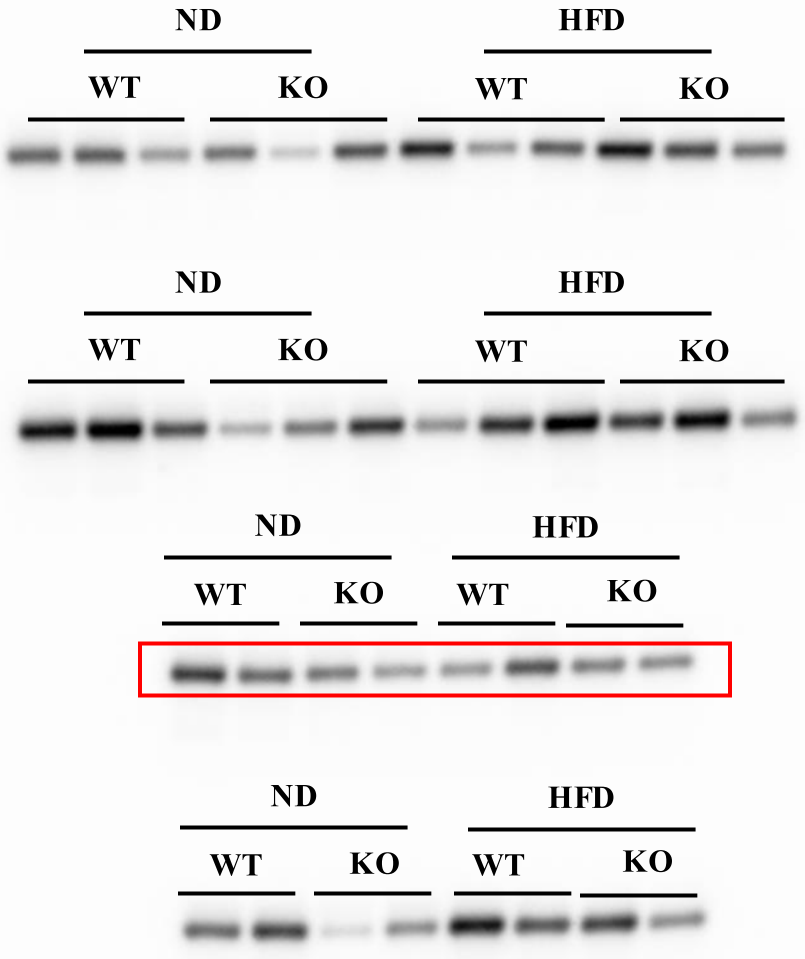

ND
HFD
WT
KO
WT
KO
ND
HFD
WT
KO
WT
KO
ND
HFD
WT
KO
WT
KO
ND
HFD
WT
KO
WT
KO

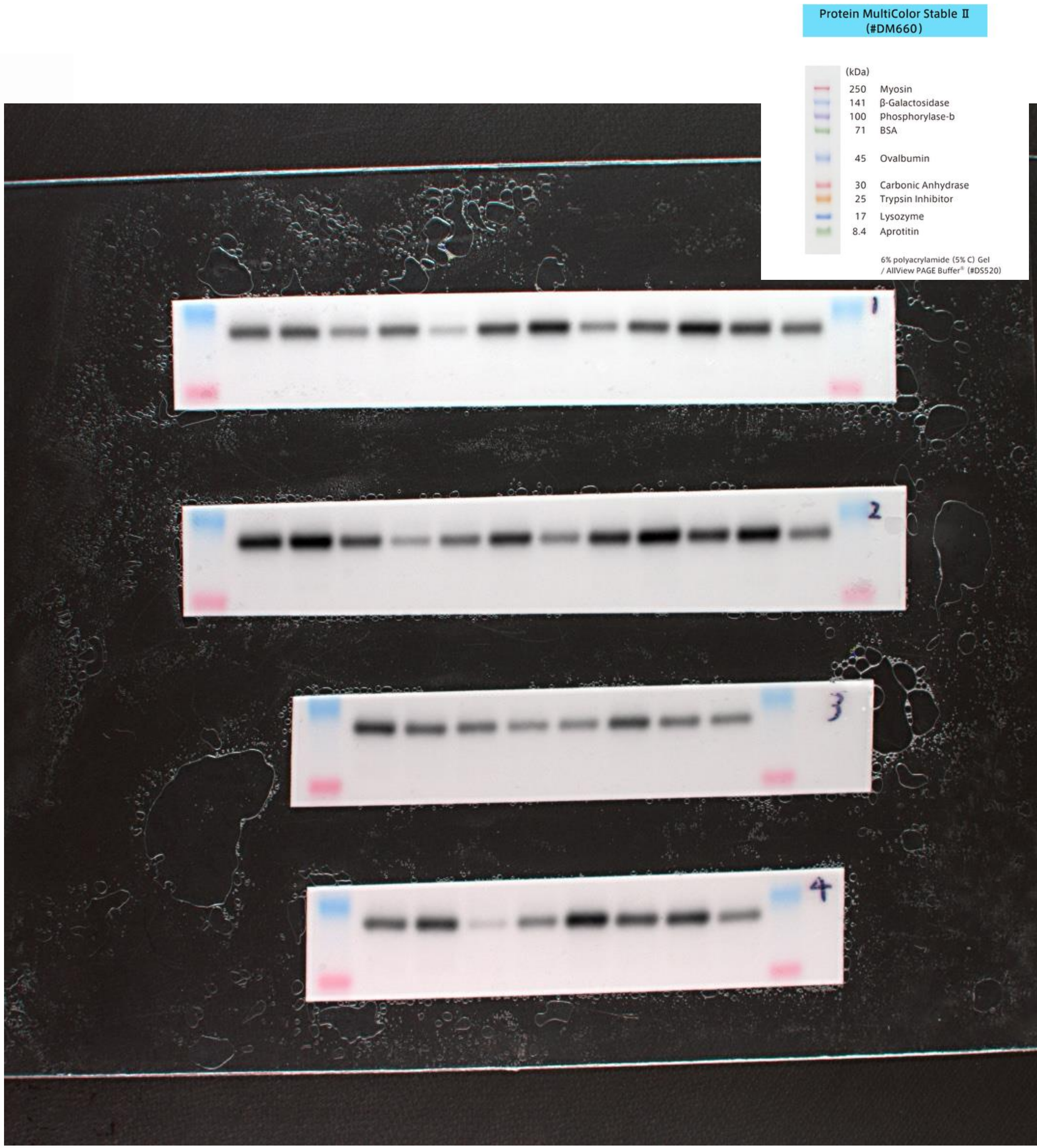

Protein MultiColor Stable II
(#DM660)
(kDa)
250 Myosin
141 β-Galactosidase
100 Phosphorylase-b
71 BSA
45 Ovalbumin
30 Carbonic Anhydrase
25 Trypsin Inhibitor
17 Lysozyme
8.4 Aprotitin
6% polyacrylamide (5% C) Gel
/ AllView PAGE Buffer® (#DS520)
1
2
3
4

p38

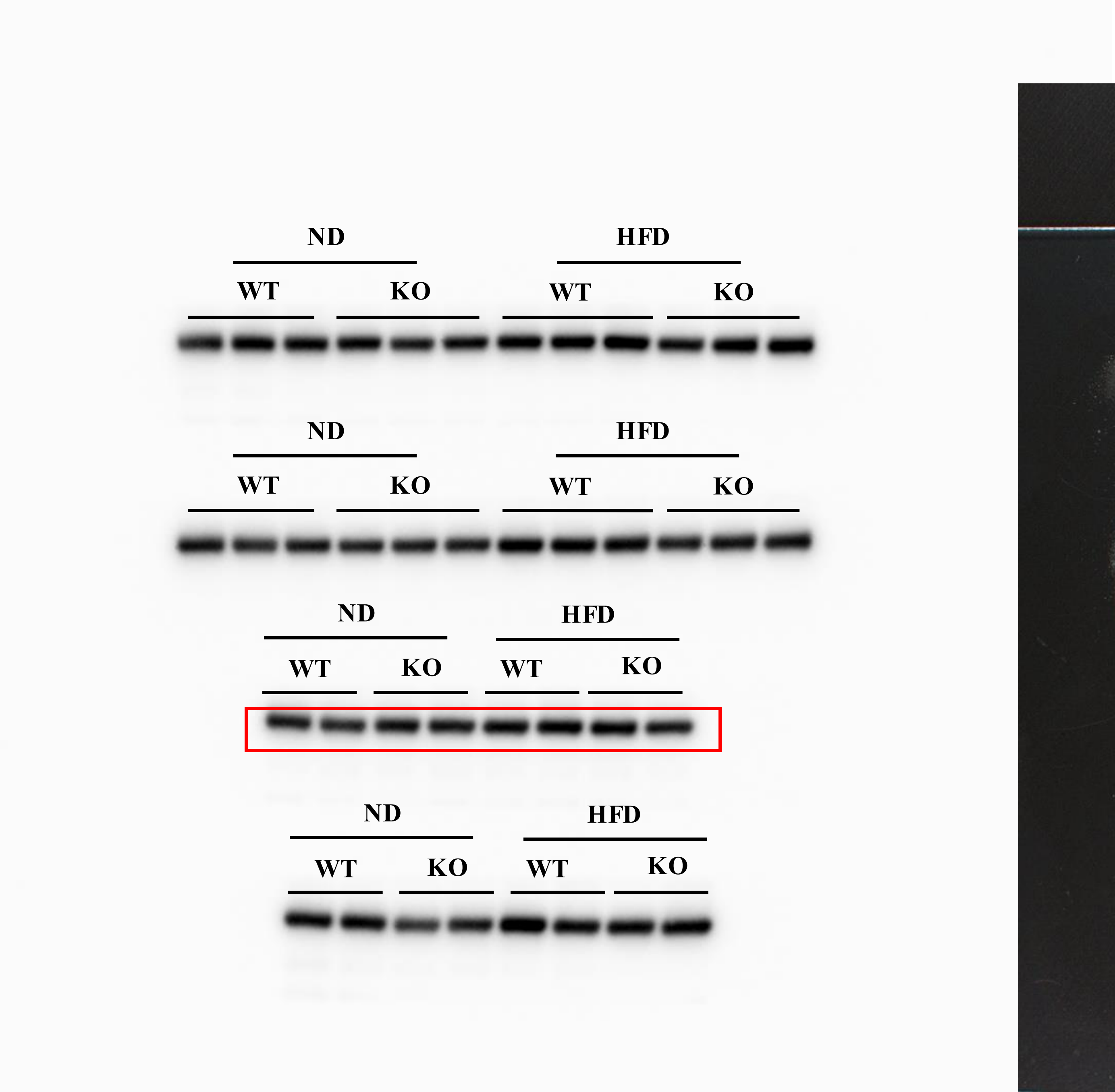
ND
HFD
WT
KO
WT
KO
ND
HFD
WT
KO
WT
KO
ND
HFD
WT
KO
WT
KO
ND
HFD
WT
KO
WT
KO

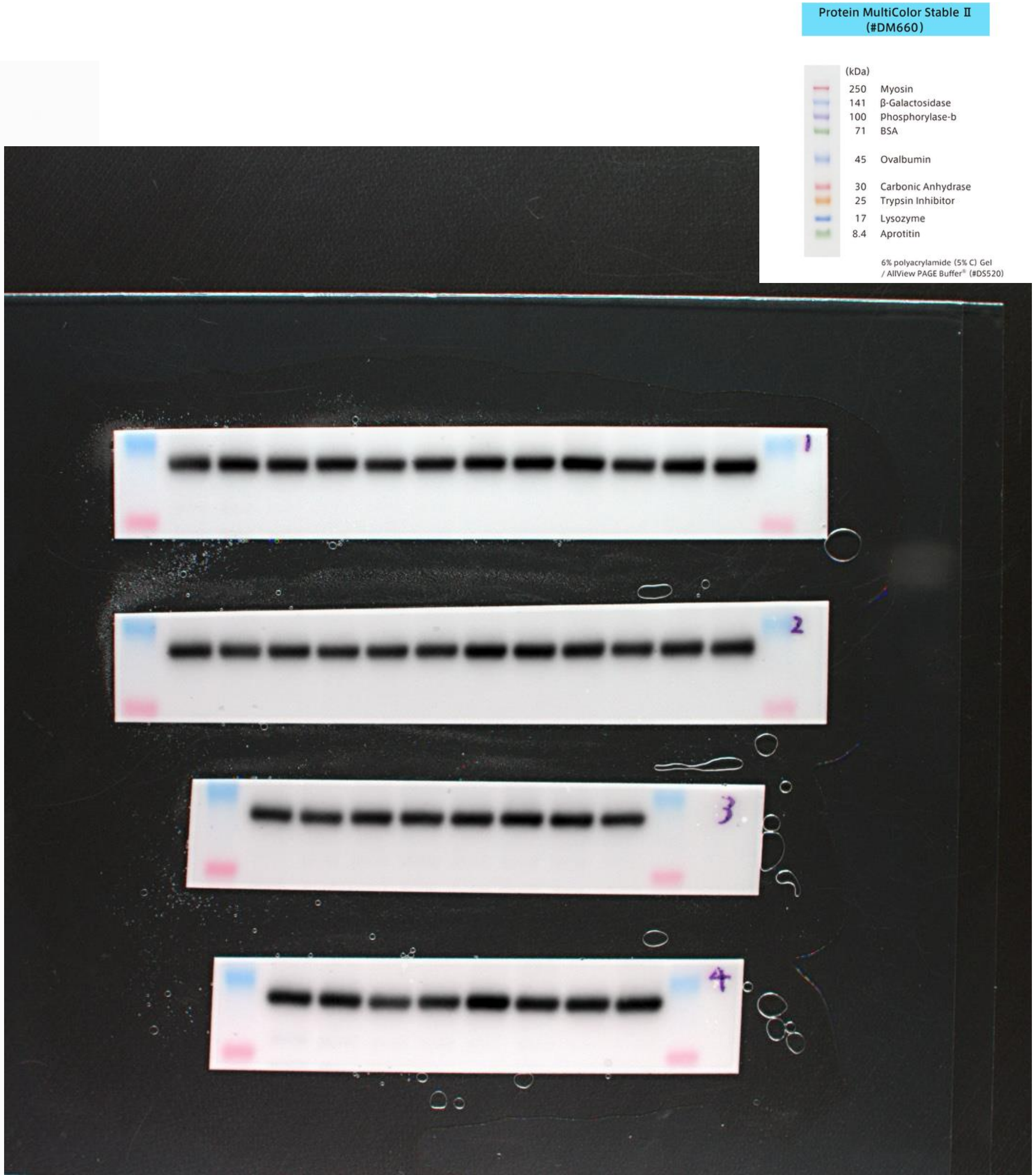
Protein MultiColor Stable II (#DM660)
(kDa)
250 Myosin
141 β-Galactosidase
100 Phosphorylase-b
71 BSA
45 Ovalbumin
30 Carbonic Anhydrase
25 Trypsin Inhibitor
17 Lysozyme
8.4 Aprotitin
6% polyacrylamide (5% C) Gel / AllView PAGE Buffer® (#DS520)
1
2
3
4

p-ERK

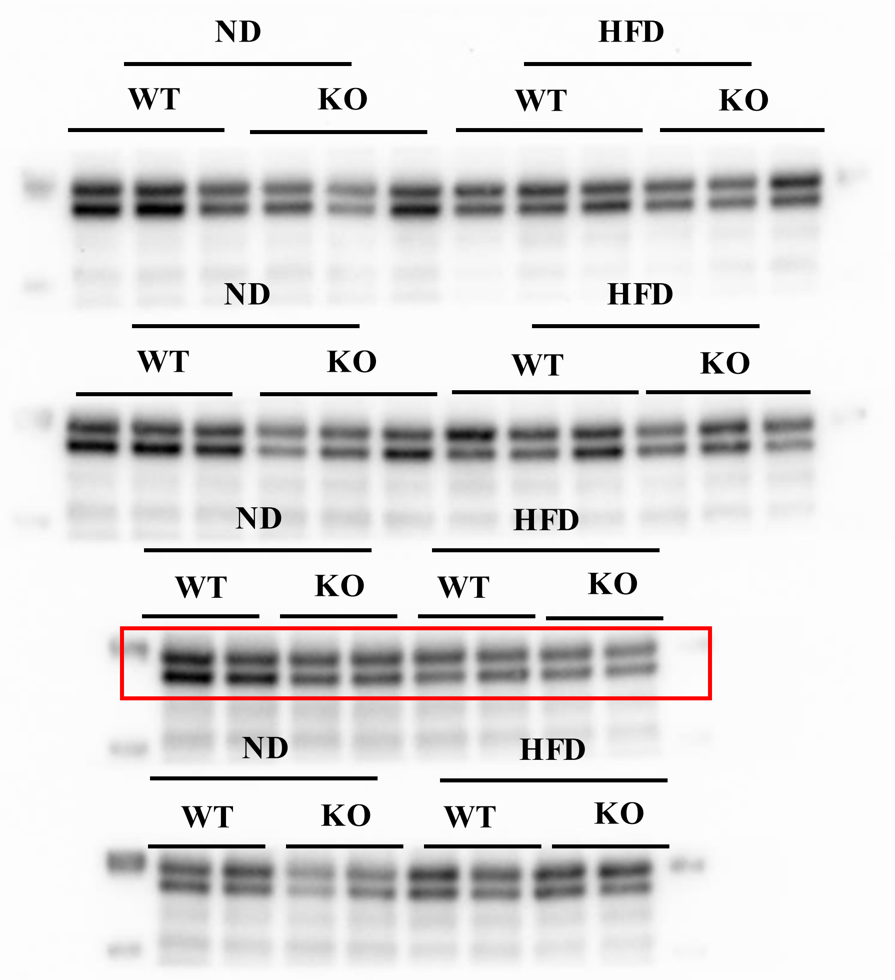
ND
HFD
WT
KO
WT
KO
ND
HFD
WT
KO
WT
KO
ND
HFD
WT
KO
WT
KO
ND
HFD
WT
KO
WT
KO

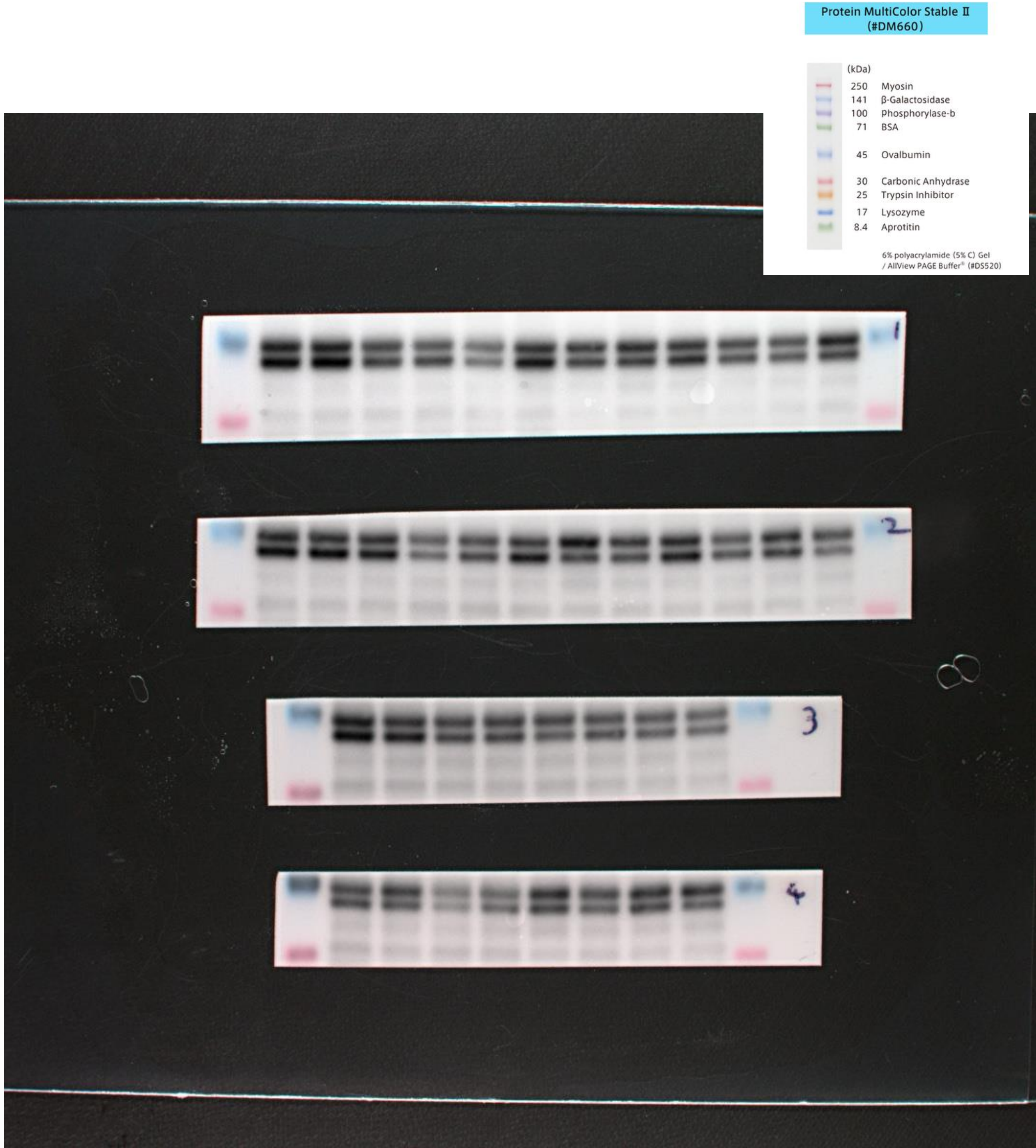
Protein MultiColor Stable Ⅱ (#DM660)
(kDa)
250 Myosin
141 β-Galactosidase
100 Phosphorylase-b
71 BSA
45 Ovalbumin
30 Carbonic Anhydrase
25 Trypsin Inhibitor
17 Lysozyme
8.4 Aprotitin
6% polyacrylamide (5% C) Gel / AllView PAGE Buffer® (#DS520)
1
2
3
4

ERK

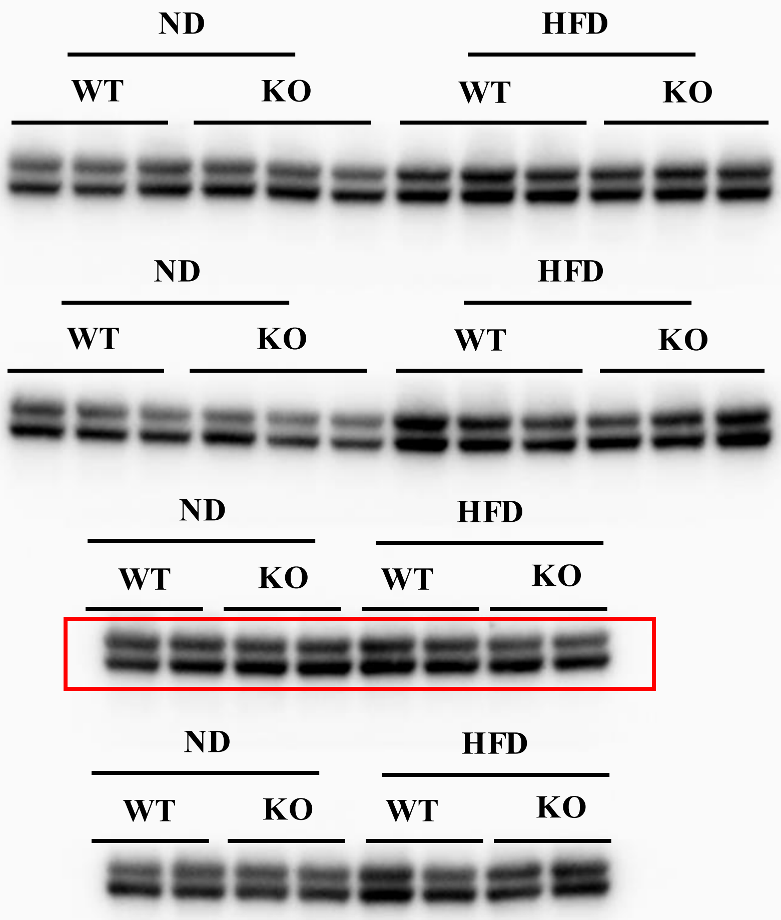
ND
HFD
WT
KO
WT
KO
ND
HFD
WT
KO
WT
KO
ND
HFD
WT
KO
WT
KO
ND
HFD
WT
KO
WT
KO

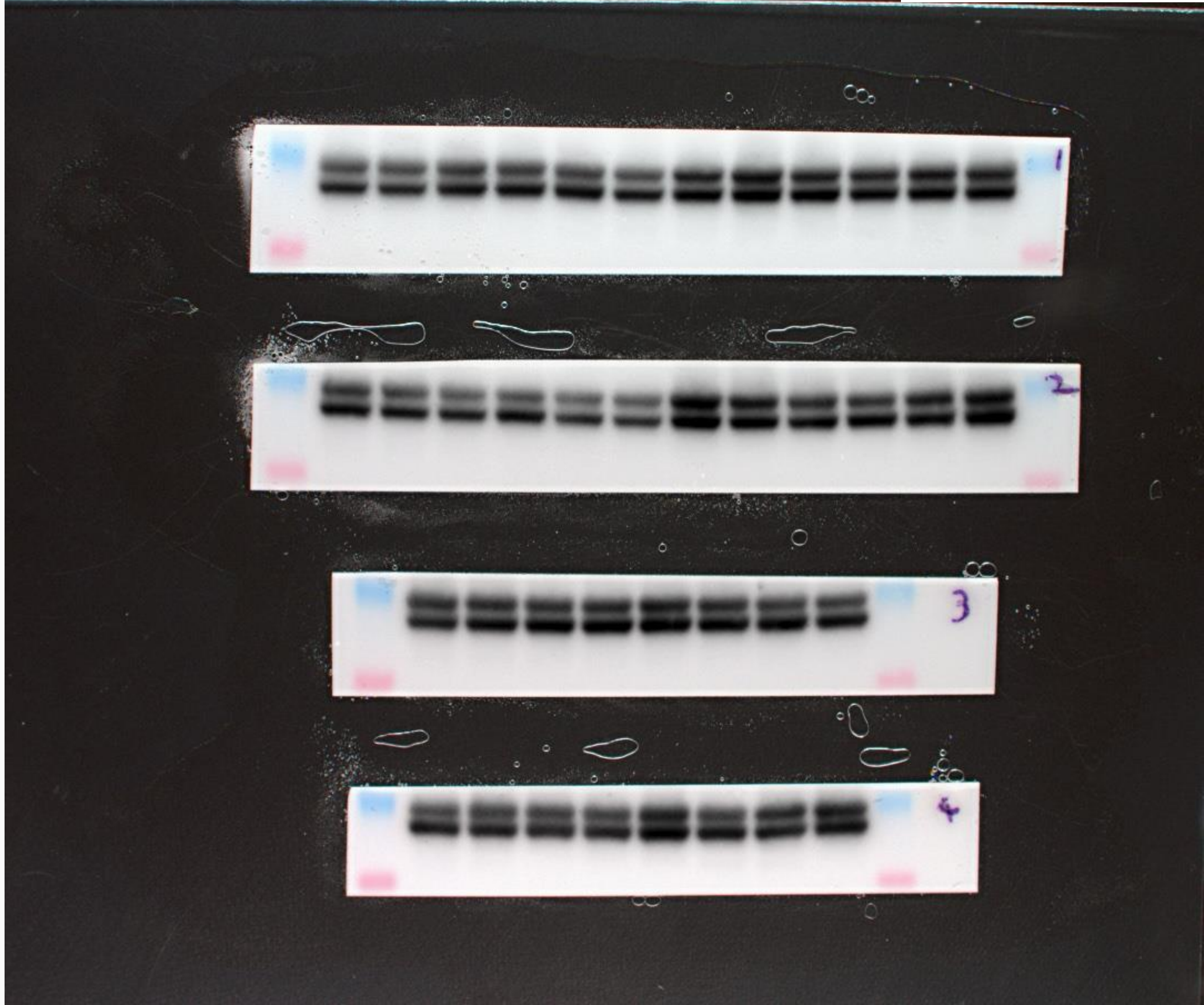
1
2
3
4

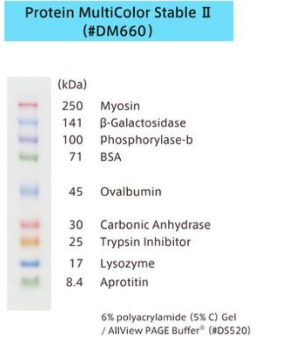
Protein MultiColor Stable II (#DM660)
(kDa)
250 Myosin
141 β-Galactosidase
100 Phosphorylase-b
71 BSA
45 Ovalbumin
30 Carbonic Anhydrase
25 Trypsin Inhibitor
17 Lysozyme
8.4 Aprotitin
6% polyacrylamide (5% C) Gel
/ AllView PAGE Buffer® (#DS520)

p-Akt(Ser)

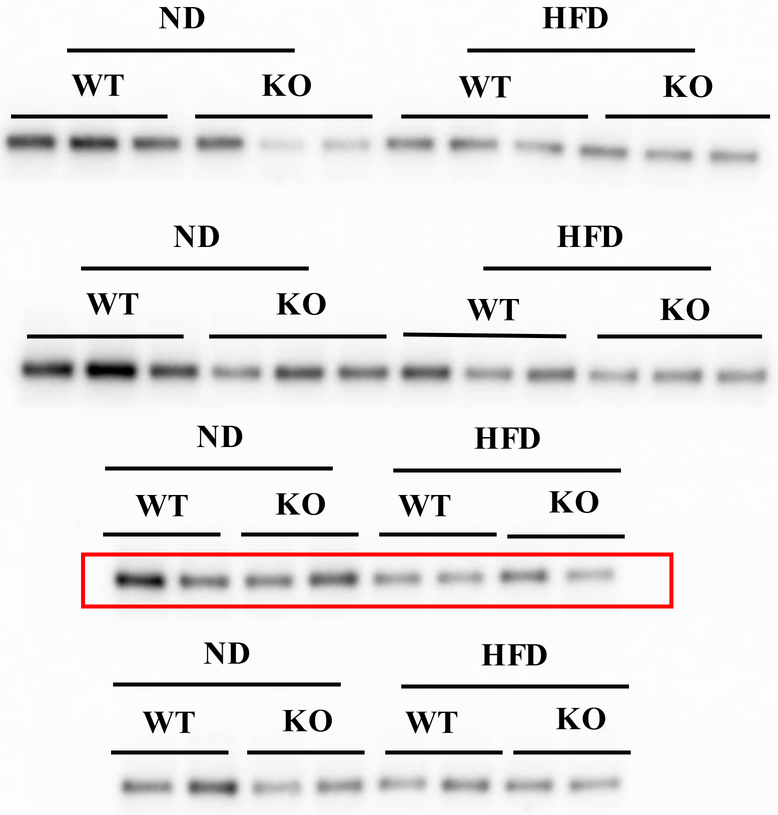
ND
HFD
WT
KO
WT
KO
ND
HFD
WT
KO
WT
KO
ND
HFD
WT
KO
WT
KO
ND
HFD
WT
KO
WT
KO

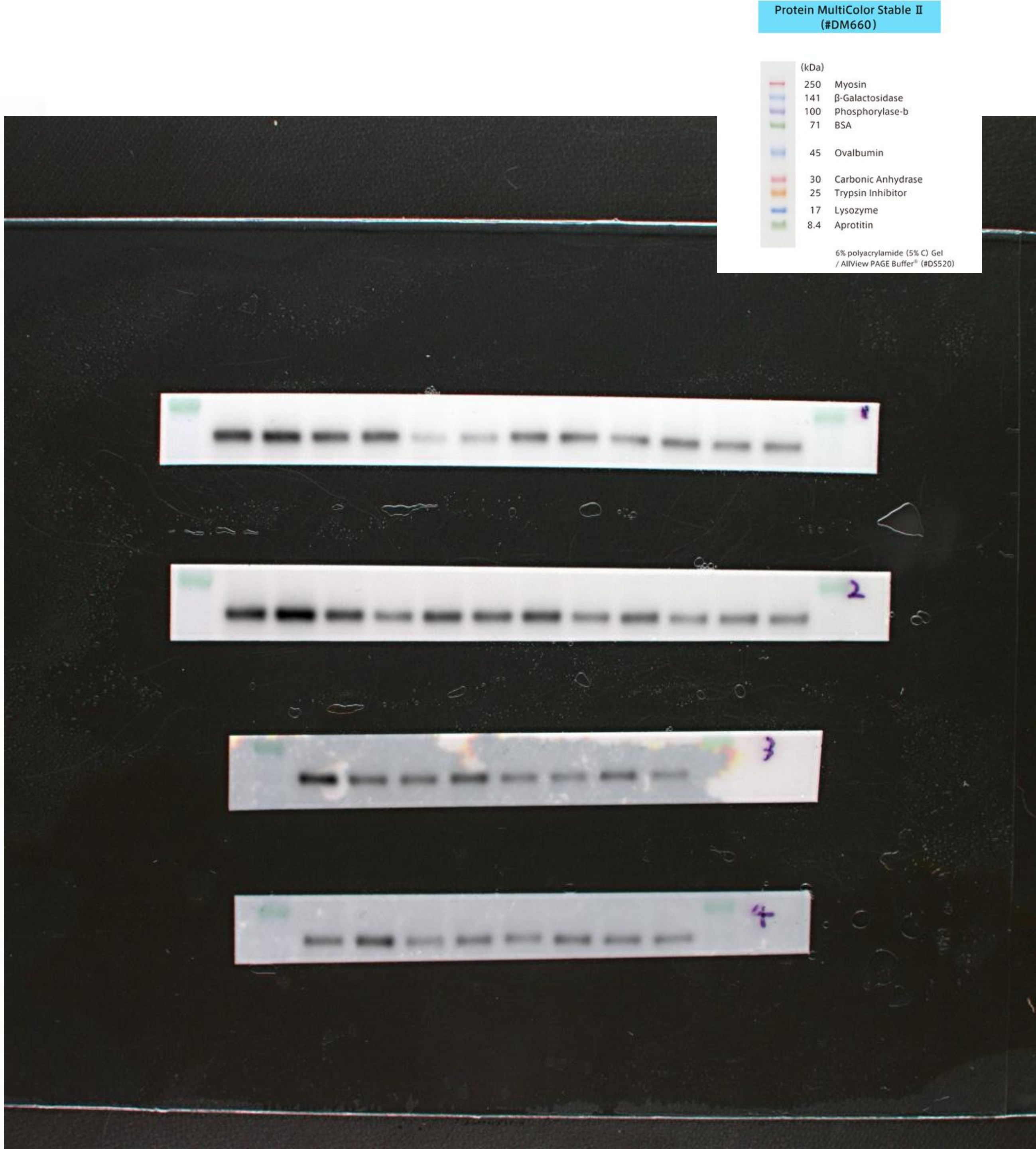
Protein MultiColor Stable Ⅱ
(#DM660)
(kDa)
250 Myosin
141 β-Galactosidase
100 Phosphorylase-b
71 BSA
45 Ovalbumin
30 Carbonic Anhydrase
25 Trypsin Inhibitor
17 Lysozyme
8.4 Aprotitin
6% polyacrylamide (5% C) Gel
/ AllView PAGE Buffer® (#DS520)
1
2
3
4

Akt

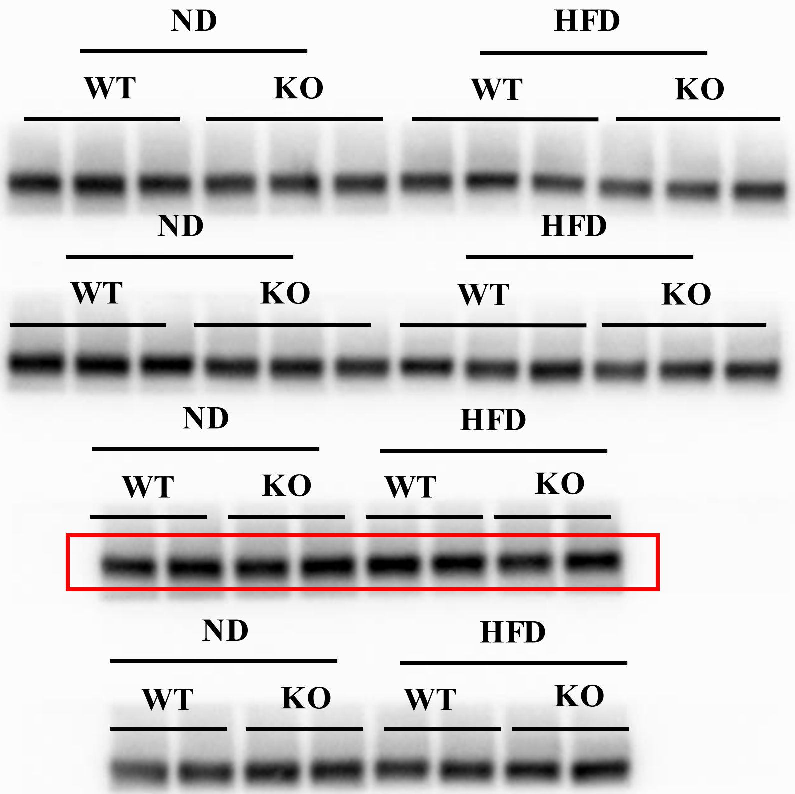
ND
HFD
WT
KO
WT
KO
ND
HFD
WT
KO
WT
KO
ND
HFD
WT
KO
WT
KO
ND
HFD
WT
KO
WT
KO

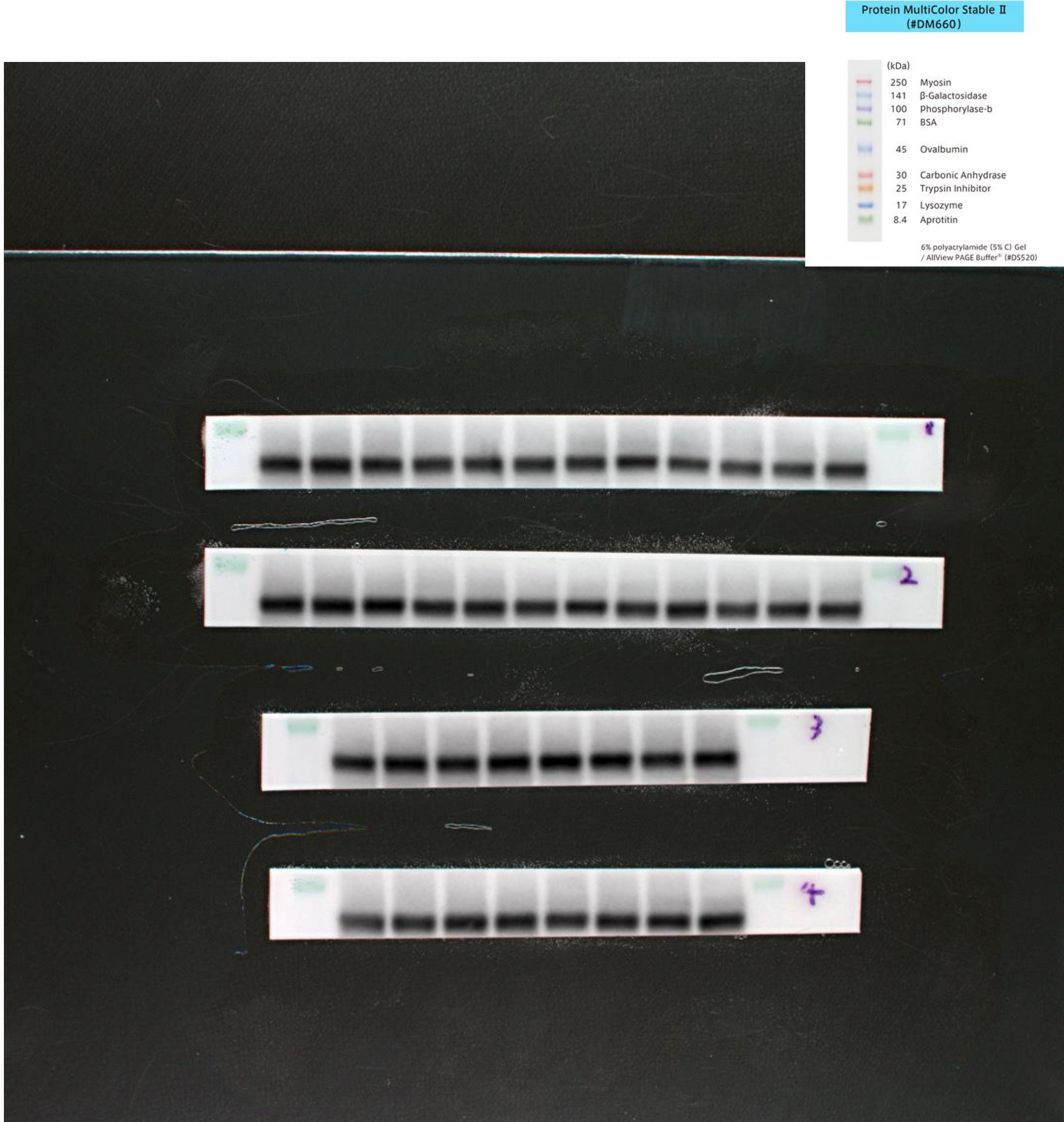
Protein MultiColor Stable Ⅱ
(#DM660)
(kDa)
250 Myosin
141 β-Galactosidase
100 Phosphorylase-b
71 BSA
45 Ovalbumin
30 Carbonic Anhydrase
25 Trypsin Inhibitor
17 Lysozyme
8.4 Aprotitin
6% polyacrylamide (5% C) Gel
/ AllView PAGE Buffer® (#DS520)
1
2
3
4
