# Supplementary material for: Renalase deficiency suppresses hepatic triglyceride accumulation in the progression to MASLD/MASH by GAN diet in male mice
Source: Physiol Rep. 2026 Jan 20;14(2):e70720. doi: 10.14814/phy2.70720 (PMC12819575; doi:10.14814/phy2.70720)
Supplement: Supplementary file 3 — Data S1. [file PHY2-14-e70720-s003.zip › PHYSREP-2025-07-681-s03.docx]

| **3way ANOVA of Fig 1d_IPGTT** |  |  |  |  |  |
| --- | --- | --- | --- | --- | --- |
|  |  |  |  |  |  |
| Source of Variation | % of total variation | P value | P value summary | Significant? |  |
| Time | 70.88 | <0.0001 | **** | Yes |  |
| Diet | 5.501 | 0.0002 | *** | Yes |  |
| Genotype | 0.0177 | 0.8156 | ns | No |  |
| Time x Diet | 0.7389 | 0.0373 | * | Yes |  |
| Time x Genotype | 0.1789 | 0.6378 | ns | No |  |
| Diet x Genotype | 0.7359 | 0.139 | ns | No |  |
| Time x Diet x Genotype | 0.6998 | 0.0464 | * | Yes |  |
|  |  |  |  |  |  |
| Row 1:WT-ND vs. Row 1:KO-ND | -1.8 | -83.19 to 79.59 | No | ns | >0.9999 |
| Row 1:WT-ND vs. Row 1:WT-GAN | -43.28 | -129.6 to 43.05 | No | ns | 0.9584 |
| Row 1:WT-ND vs. Row 1:KO-GAN | -35.6 | -117.0 to 45.79 | No | ns | 0.9899 |
| Row 1:WT-ND vs. Row 2:WT-ND | -224.6 | -287.1 to -162.1 | Yes | **** | <0.0001 |
| Row 1:WT-ND vs. Row 2:KO-ND | -165.9 | -247.3 to -84.51 | Yes | **** | <0.0001 |
| Row 1:WT-ND vs. Row 2:WT-GAN | -245.7 | -332.0 to -159.3 | Yes | **** | <0.0001 |
| Row 1:WT-ND vs. Row 2:KO-GAN | -264.9 | -346.3 to -183.5 | Yes | **** | <0.0001 |
| Row 1:WT-ND vs. Row 3:WT-ND | -196.2 | -258.7 to -133.7 | Yes | **** | <0.0001 |
| Row 1:WT-ND vs. Row 3:KO-ND | -185.5 | -266.9 to -104.1 | Yes | **** | <0.0001 |
| Row 1:WT-ND vs. Row 3:WT-GAN | -254.3 | -340.6 to -167.9 | Yes | **** | <0.0001 |
| Row 1:WT-ND vs. Row 3:KO-GAN | -278.2 | -359.6 to -196.8 | Yes | **** | <0.0001 |
| Row 1:WT-ND vs. Row 4:WT-ND | -135.8 | -198.3 to -73.33 | Yes | **** | <0.0001 |
| Row 1:WT-ND vs. Row 4:KO-ND | -99.4 | -180.8 to -18.01 | Yes | ** | 0.003 |
| Row 1:WT-ND vs. Row 4:WT-GAN | -151.5 | -237.9 to -65.20 | Yes | **** | <0.0001 |
| Row 1:WT-ND vs. Row 4:KO-GAN | -189.7 | -271.1 to -108.3 | Yes | **** | <0.0001 |
| Row 1:WT-ND vs. Row 5:WT-ND | -25.2 | -87.67 to 37.27 | No | ns | 0.9957 |
| Row 1:WT-ND vs. Row 5:KO-ND | -24 | -105.4 to 57.39 | No | ns | >0.9999 |
| Row 1:WT-ND vs. Row 5:WT-GAN | -45.03 | -131.4 to 41.30 | No | ns | 0.9401 |
| Row 1:WT-ND vs. Row 5:KO-GAN | -48.3 | -129.7 to 33.09 | No | ns | 0.8327 |
| Row 1:KO-ND vs. Row 1:WT-GAN | -41.48 | -127.8 to 44.85 | No | ns | 0.9726 |
| Row 1:KO-ND vs. Row 1:KO-GAN | -33.8 | -115.2 to 47.59 | No | ns | 0.9945 |
| Row 1:KO-ND vs. Row 2:WT-ND | -222.8 | -304.2 to -141.4 | Yes | **** | <0.0001 |
| Row 1:KO-ND vs. Row 2:KO-ND | -164.1 | -226.6 to -101.6 | Yes | **** | <0.0001 |
| Row 1:KO-ND vs. Row 2:WT-GAN | -243.9 | -330.2 to -157.5 | Yes | **** | <0.0001 |
| Row 1:KO-ND vs. Row 2:KO-GAN | -263.1 | -344.5 to -181.7 | Yes | **** | <0.0001 |
| Row 1:KO-ND vs. Row 3:WT-ND | -194.4 | -275.8 to -113.0 | Yes | **** | <0.0001 |
| Row 1:KO-ND vs. Row 3:KO-ND | -183.7 | -246.2 to -121.2 | Yes | **** | <0.0001 |
| Row 1:KO-ND vs. Row 3:WT-GAN | -252.5 | -338.8 to -166.1 | Yes | **** | <0.0001 |
| Row 1:KO-ND vs. Row 3:KO-GAN | -276.4 | -357.8 to -195.0 | Yes | **** | <0.0001 |
| Row 1:KO-ND vs. Row 4:WT-ND | -134 | -215.4 to -52.61 | Yes | **** | <0.0001 |
| Row 1:KO-ND vs. Row 4:KO-ND | -97.6 | -160.1 to -35.13 | Yes | **** | <0.0001 |
| Row 1:KO-ND vs. Row 4:WT-GAN | -149.7 | -236.1 to -63.40 | Yes | **** | <0.0001 |
| Row 1:KO-ND vs. Row 4:KO-GAN | -187.9 | -269.3 to -106.5 | Yes | **** | <0.0001 |
| Row 1:KO-ND vs. Row 5:WT-ND | -23.4 | -104.8 to 57.99 | No | ns | >0.9999 |
| Row 1:KO-ND vs. Row 5:KO-ND | -22.2 | -84.67 to 40.27 | No | ns | 0.9992 |
| Row 1:KO-ND vs. Row 5:WT-GAN | -43.22 | -129.6 to 43.10 | No | ns | 0.9589 |
| Row 1:KO-ND vs. Row 5:KO-GAN | -46.5 | -127.9 to 34.89 | No | ns | 0.8731 |
| Row 1:WT-GAN vs. Row 1:KO-GAN | 7.675 | -78.65 to 94.00 | No | ns | >0.9999 |
| Row 1:WT-GAN vs. Row 2:WT-ND | -181.3 | -267.7 to -95.00 | Yes | **** | <0.0001 |
| Row 1:WT-GAN vs. Row 2:KO-ND | -122.6 | -209.0 to -36.30 | Yes | *** | 0.0001 |
| Row 1:WT-GAN vs. Row 2:WT-GAN | -202.4 | -272.2 to -132.5 | Yes | **** | <0.0001 |
| Row 1:WT-GAN vs. Row 2:KO-GAN | -221.6 | -308.0 to -135.3 | Yes | **** | <0.0001 |
| Row 1:WT-GAN vs. Row 3:WT-ND | -152.9 | -239.3 to -66.60 | Yes | **** | <0.0001 |
| Row 1:WT-GAN vs. Row 3:KO-ND | -142.2 | -228.6 to -55.90 | Yes | **** | <0.0001 |
| Row 1:WT-GAN vs. Row 3:WT-GAN | -211 | -280.8 to -141.2 | Yes | **** | <0.0001 |
| Row 1:WT-GAN vs. Row 3:KO-GAN | -234.9 | -321.3 to -148.6 | Yes | **** | <0.0001 |
| Row 1:WT-GAN vs. Row 4:WT-ND | -92.53 | -178.9 to -6.199 | Yes | * | 0.0218 |
| Row 1:WT-GAN vs. Row 4:KO-ND | -56.13 | -142.5 to 30.20 | No | ns | 0.7032 |
| Row 1:WT-GAN vs. Row 4:WT-GAN | -108.3 | -178.1 to -38.41 | Yes | **** | <0.0001 |
| Row 1:WT-GAN vs. Row 4:KO-GAN | -146.4 | -232.8 to -60.10 | Yes | **** | <0.0001 |
| Row 1:WT-GAN vs. Row 5:WT-ND | 18.08 | -68.25 to 104.4 | No | ns | >0.9999 |
| Row 1:WT-GAN vs. Row 5:KO-ND | 19.28 | -67.05 to 105.6 | No | ns | >0.9999 |
| Row 1:WT-GAN vs. Row 5:WT-GAN | -1.75 | -71.59 to 68.09 | No | ns | >0.9999 |
| Row 1:WT-GAN vs. Row 5:KO-GAN | -5.025 | -91.35 to 81.30 | No | ns | >0.9999 |
| Row 1:KO-GAN vs. Row 2:WT-ND | -189 | -270.4 to -107.6 | Yes | **** | <0.0001 |
| Row 1:KO-GAN vs. Row 2:KO-ND | -130.3 | -211.7 to -48.91 | Yes | **** | <0.0001 |
| Row 1:KO-GAN vs. Row 2:WT-GAN | -210.1 | -296.4 to -123.7 | Yes | **** | <0.0001 |
| Row 1:KO-GAN vs. Row 2:KO-GAN | -229.3 | -291.8 to -166.8 | Yes | **** | <0.0001 |
| Row 1:KO-GAN vs. Row 3:WT-ND | -160.6 | -242.0 to -79.21 | Yes | **** | <0.0001 |
| Row 1:KO-GAN vs. Row 3:KO-ND | -149.9 | -231.3 to -68.51 | Yes | **** | <0.0001 |
| Row 1:KO-GAN vs. Row 3:WT-GAN | -218.7 | -305.0 to -132.3 | Yes | **** | <0.0001 |
| Row 1:KO-GAN vs. Row 3:KO-GAN | -242.6 | -305.1 to -180.1 | Yes | **** | <0.0001 |
| Row 1:KO-GAN vs. Row 4:WT-ND | -100.2 | -181.6 to -18.81 | Yes | ** | 0.0027 |
| Row 1:KO-GAN vs. Row 4:KO-ND | -63.8 | -145.2 to 17.59 | No | ns | 0.3504 |
| Row 1:KO-GAN vs. Row 4:WT-GAN | -115.9 | -202.3 to -29.60 | Yes | *** | 0.0005 |
| Row 1:KO-GAN vs. Row 4:KO-GAN | -154.1 | -216.6 to -91.63 | Yes | **** | <0.0001 |
| Row 1:KO-GAN vs. Row 5:WT-ND | 10.4 | -70.99 to 91.79 | No | ns | >0.9999 |
| Row 1:KO-GAN vs. Row 5:KO-ND | 11.6 | -69.79 to 92.99 | No | ns | >0.9999 |
| Row 1:KO-GAN vs. Row 5:WT-GAN | -9.425 | -95.75 to 76.90 | No | ns | >0.9999 |
| Row 1:KO-GAN vs. Row 5:KO-GAN | -12.7 | -75.17 to 49.77 | No | ns | >0.9999 |
| Row 2:WT-ND vs. Row 2:KO-ND | 58.7 | -22.69 to 140.1 | No | ns | 0.5119 |
| Row 2:WT-ND vs. Row 2:WT-GAN | -21.05 | -107.4 to 65.28 | No | ns | >0.9999 |
| Row 2:WT-ND vs. Row 2:KO-GAN | -40.3 | -121.7 to 41.09 | No | ns | 0.9631 |
| Row 2:WT-ND vs. Row 3:WT-ND | 28.4 | -34.07 to 90.87 | No | ns | 0.9834 |
| Row 2:WT-ND vs. Row 3:KO-ND | 39.1 | -42.29 to 120.5 | No | ns | 0.9726 |
| Row 2:WT-ND vs. Row 3:WT-GAN | -29.68 | -116.0 to 56.65 | No | ns | 0.9995 |
| Row 2:WT-ND vs. Row 3:KO-GAN | -53.6 | -135.0 to 27.79 | No | ns | 0.6815 |
| Row 2:WT-ND vs. Row 4:WT-ND | 88.8 | 26.33 to 151.3 | Yes | *** | 0.0002 |
| Row 2:WT-ND vs. Row 4:KO-ND | 125.2 | 43.81 to 206.6 | Yes | **** | <0.0001 |
| Row 2:WT-ND vs. Row 4:WT-GAN | 73.08 | -13.25 to 159.4 | No | ns | 0.2195 |
| Row 2:WT-ND vs. Row 4:KO-GAN | 34.9 | -46.49 to 116.3 | No | ns | 0.992 |
| Row 2:WT-ND vs. Row 5:WT-ND | 199.4 | 136.9 to 261.9 | Yes | **** | <0.0001 |
| Row 2:WT-ND vs. Row 5:KO-ND | 200.6 | 119.2 to 282.0 | Yes | **** | <0.0001 |
| Row 2:WT-ND vs. Row 5:WT-GAN | 179.6 | 93.25 to 265.9 | Yes | **** | <0.0001 |
| Row 2:WT-ND vs. Row 5:KO-GAN | 176.3 | 94.91 to 257.7 | Yes | **** | <0.0001 |
| Row 2:KO-ND vs. Row 2:WT-GAN | -79.75 | -166.1 to 6.576 | No | ns | 0.1101 |
| Row 2:KO-ND vs. Row 2:KO-GAN | -99 | -180.4 to -17.61 | Yes | ** | 0.0033 |
| Row 2:KO-ND vs. Row 3:WT-ND | -30.3 | -111.7 to 51.09 | No | ns | 0.9986 |
| Row 2:KO-ND vs. Row 3:KO-ND | -19.6 | -82.07 to 42.87 | No | ns | 0.9999 |
| Row 2:KO-ND vs. Row 3:WT-GAN | -88.38 | -174.7 to -2.049 | Yes | * | 0.0383 |
| Row 2:KO-ND vs. Row 3:KO-GAN | -112.3 | -193.7 to -30.91 | Yes | *** | 0.0003 |
| Row 2:KO-ND vs. Row 4:WT-ND | 30.1 | -51.29 to 111.5 | No | ns | 0.9987 |
| Row 2:KO-ND vs. Row 4:KO-ND | 66.5 | 4.035 to 129.0 | Yes | * | 0.024 |
| Row 2:KO-ND vs. Row 4:WT-GAN | 14.38 | -71.95 to 100.7 | No | ns | >0.9999 |
| Row 2:KO-ND vs. Row 4:KO-GAN | -23.8 | -105.2 to 57.59 | No | ns | >0.9999 |
| Row 2:KO-ND vs. Row 5:WT-ND | 140.7 | 59.31 to 222.1 | Yes | **** | <0.0001 |
| Row 2:KO-ND vs. Row 5:KO-ND | 141.9 | 79.43 to 204.4 | Yes | **** | <0.0001 |
| Row 2:KO-ND vs. Row 5:WT-GAN | 120.9 | 34.55 to 207.2 | Yes | *** | 0.0002 |
| Row 2:KO-ND vs. Row 5:KO-GAN | 117.6 | 36.21 to 199.0 | Yes | **** | <0.0001 |
| Row 2:WT-GAN vs. Row 2:KO-GAN | -19.25 | -105.6 to 67.08 | No | ns | >0.9999 |
| Row 2:WT-GAN vs. Row 3:WT-ND | 49.45 | -36.88 to 135.8 | No | ns | 0.8706 |
| Row 2:WT-GAN vs. Row 3:KO-ND | 60.15 | -26.18 to 146.5 | No | ns | 0.5788 |
| Row 2:WT-GAN vs. Row 3:WT-GAN | -8.625 | -78.46 to 61.21 | No | ns | >0.9999 |
| Row 2:WT-GAN vs. Row 3:KO-GAN | -32.55 | -118.9 to 53.78 | No | ns | 0.9983 |
| Row 2:WT-GAN vs. Row 4:WT-ND | 109.9 | 23.52 to 196.2 | Yes | ** | 0.0015 |
| Row 2:WT-GAN vs. Row 4:KO-ND | 146.3 | 59.92 to 232.6 | Yes | **** | <0.0001 |
| Row 2:WT-GAN vs. Row 4:WT-GAN | 94.13 | 24.29 to 164.0 | Yes | *** | 0.0005 |
| Row 2:WT-GAN vs. Row 4:KO-GAN | 55.95 | -30.38 to 142.3 | No | ns | 0.7083 |
| Row 2:WT-GAN vs. Row 5:WT-ND | 220.5 | 134.1 to 306.8 | Yes | **** | <0.0001 |
| Row 2:WT-GAN vs. Row 5:KO-ND | 221.7 | 135.3 to 308.0 | Yes | **** | <0.0001 |
| Row 2:WT-GAN vs. Row 5:WT-GAN | 200.6 | 130.8 to 270.5 | Yes | **** | <0.0001 |
| Row 2:WT-GAN vs. Row 5:KO-GAN | 197.4 | 111.0 to 283.7 | Yes | **** | <0.0001 |
| Row 2:KO-GAN vs. Row 3:WT-ND | 68.7 | -12.69 to 150.1 | No | ns | 0.2238 |
| Row 2:KO-GAN vs. Row 3:KO-ND | 79.4 | -1.989 to 160.8 | No | ns | 0.0651 |
| Row 2:KO-GAN vs. Row 3:WT-GAN | 10.63 | -75.70 to 96.95 | No | ns | >0.9999 |
| Row 2:KO-GAN vs. Row 3:KO-GAN | -13.3 | -75.77 to 49.17 | No | ns | >0.9999 |
| Row 2:KO-GAN vs. Row 4:WT-ND | 129.1 | 47.71 to 210.5 | Yes | **** | <0.0001 |
| Row 2:KO-GAN vs. Row 4:KO-ND | 165.5 | 84.11 to 246.9 | Yes | **** | <0.0001 |
| Row 2:KO-GAN vs. Row 4:WT-GAN | 113.4 | 27.05 to 199.7 | Yes | *** | 0.0008 |
| Row 2:KO-GAN vs. Row 4:KO-GAN | 75.2 | 12.73 to 137.7 | Yes | ** | 0.0041 |
| Row 2:KO-GAN vs. Row 5:WT-ND | 239.7 | 158.3 to 321.1 | Yes | **** | <0.0001 |
| Row 2:KO-GAN vs. Row 5:KO-ND | 240.9 | 159.5 to 322.3 | Yes | **** | <0.0001 |
| Row 2:KO-GAN vs. Row 5:WT-GAN | 219.9 | 133.5 to 306.2 | Yes | **** | <0.0001 |
| Row 2:KO-GAN vs. Row 5:KO-GAN | 216.6 | 154.1 to 279.1 | Yes | **** | <0.0001 |
| Row 3:WT-ND vs. Row 3:KO-ND | 10.7 | -70.69 to 92.09 | No | ns | >0.9999 |
| Row 3:WT-ND vs. Row 3:WT-GAN | -58.08 | -144.4 to 28.25 | No | ns | 0.644 |
| Row 3:WT-ND vs. Row 3:KO-GAN | -82 | -163.4 to -0.6114 | Yes | * | 0.046 |
| Row 3:WT-ND vs. Row 4:WT-ND | 60.4 | -2.065 to 122.9 | No | ns | 0.071 |
| Row 3:WT-ND vs. Row 4:KO-ND | 96.8 | 15.41 to 178.2 | Yes | ** | 0.0048 |
| Row 3:WT-ND vs. Row 4:WT-GAN | 44.68 | -41.65 to 131.0 | No | ns | 0.9442 |
| Row 3:WT-ND vs. Row 4:KO-GAN | 6.5 | -74.89 to 87.89 | No | ns | >0.9999 |
| Row 3:WT-ND vs. Row 5:WT-ND | 171 | 108.5 to 233.5 | Yes | **** | <0.0001 |
| Row 3:WT-ND vs. Row 5:KO-ND | 172.2 | 90.81 to 253.6 | Yes | **** | <0.0001 |
| Row 3:WT-ND vs. Row 5:WT-GAN | 151.2 | 64.85 to 237.5 | Yes | **** | <0.0001 |
| Row 3:WT-ND vs. Row 5:KO-GAN | 147.9 | 66.51 to 229.3 | Yes | **** | <0.0001 |
| Row 3:KO-ND vs. Row 3:WT-GAN | -68.78 | -155.1 to 17.55 | No | ns | 0.3207 |
| Row 3:KO-ND vs. Row 3:KO-GAN | -92.7 | -174.1 to -11.31 | Yes | ** | 0.0093 |
| Row 3:KO-ND vs. Row 4:WT-ND | 49.7 | -31.69 to 131.1 | No | ns | 0.797 |
| Row 3:KO-ND vs. Row 4:KO-ND | 86.1 | 23.63 to 148.6 | Yes | *** | 0.0003 |
| Row 3:KO-ND vs. Row 4:WT-GAN | 33.98 | -52.35 to 120.3 | No | ns | 0.9971 |
| Row 3:KO-ND vs. Row 4:KO-GAN | -4.2 | -85.59 to 77.19 | No | ns | >0.9999 |
| Row 3:KO-ND vs. Row 5:WT-ND | 160.3 | 78.91 to 241.7 | Yes | **** | <0.0001 |
| Row 3:KO-ND vs. Row 5:KO-ND | 161.5 | 99.03 to 224.0 | Yes | **** | <0.0001 |
| Row 3:KO-ND vs. Row 5:WT-GAN | 140.5 | 54.15 to 226.8 | Yes | **** | <0.0001 |
| Row 3:KO-ND vs. Row 5:KO-GAN | 137.2 | 55.81 to 218.6 | Yes | **** | <0.0001 |
| Row 3:WT-GAN vs. Row 3:KO-GAN | -23.93 | -110.3 to 62.40 | No | ns | >0.9999 |
| Row 3:WT-GAN vs. Row 4:WT-ND | 118.5 | 32.15 to 204.8 | Yes | *** | 0.0003 |
| Row 3:WT-GAN vs. Row 4:KO-ND | 154.9 | 68.55 to 241.2 | Yes | **** | <0.0001 |
| Row 3:WT-GAN vs. Row 4:WT-GAN | 102.8 | 32.91 to 172.6 | Yes | **** | <0.0001 |
| Row 3:WT-GAN vs. Row 4:KO-GAN | 64.58 | -21.75 to 150.9 | No | ns | 0.4402 |
| Row 3:WT-GAN vs. Row 5:WT-ND | 229.1 | 142.7 to 315.4 | Yes | **** | <0.0001 |
| Row 3:WT-GAN vs. Row 5:KO-ND | 230.3 | 143.9 to 316.6 | Yes | **** | <0.0001 |
| Row 3:WT-GAN vs. Row 5:WT-GAN | 209.3 | 139.4 to 279.1 | Yes | **** | <0.0001 |
| Row 3:WT-GAN vs. Row 5:KO-GAN | 206 | 119.6 to 292.3 | Yes | **** | <0.0001 |
| Row 3:KO-GAN vs. Row 4:WT-ND | 142.4 | 61.01 to 223.8 | Yes | **** | <0.0001 |
| Row 3:KO-GAN vs. Row 4:KO-ND | 178.8 | 97.41 to 260.2 | Yes | **** | <0.0001 |
| Row 3:KO-GAN vs. Row 4:WT-GAN | 126.7 | 40.35 to 213.0 | Yes | **** | <0.0001 |
| Row 3:KO-GAN vs. Row 4:KO-GAN | 88.5 | 26.03 to 151.0 | Yes | *** | 0.0002 |
| Row 3:KO-GAN vs. Row 5:WT-ND | 253 | 171.6 to 334.4 | Yes | **** | <0.0001 |
| Row 3:KO-GAN vs. Row 5:KO-ND | 254.2 | 172.8 to 335.6 | Yes | **** | <0.0001 |
| Row 3:KO-GAN vs. Row 5:WT-GAN | 233.2 | 146.8 to 319.5 | Yes | **** | <0.0001 |
| Row 3:KO-GAN vs. Row 5:KO-GAN | 229.9 | 167.4 to 292.4 | Yes | **** | <0.0001 |
| Row 4:WT-ND vs. Row 4:KO-ND | 36.4 | -44.99 to 117.8 | No | ns | 0.9871 |
| Row 4:WT-ND vs. Row 4:WT-GAN | -15.73 | -102.1 to 70.60 | No | ns | >0.9999 |
| Row 4:WT-ND vs. Row 4:KO-GAN | -53.9 | -135.3 to 27.49 | No | ns | 0.6718 |
| Row 4:WT-ND vs. Row 5:WT-ND | 110.6 | 48.13 to 173.1 | Yes | **** | <0.0001 |
| Row 4:WT-ND vs. Row 5:KO-ND | 111.8 | 30.41 to 193.2 | Yes | *** | 0.0003 |
| Row 4:WT-ND vs. Row 5:WT-GAN | 90.78 | 4.449 to 177.1 | Yes | * | 0.0277 |
| Row 4:WT-ND vs. Row 5:KO-GAN | 87.5 | 6.111 to 168.9 | Yes | * | 0.0209 |
| Row 4:KO-ND vs. Row 4:WT-GAN | -52.13 | -138.5 to 34.20 | No | ns | 0.8116 |
| Row 4:KO-ND vs. Row 4:KO-GAN | -90.3 | -171.7 to -8.911 | Yes | * | 0.0136 |
| Row 4:KO-ND vs. Row 5:WT-ND | 74.2 | -7.189 to 155.6 | No | ns | 0.1236 |
| Row 4:KO-ND vs. Row 5:KO-ND | 75.4 | 12.93 to 137.9 | Yes | ** | 0.0039 |
| Row 4:KO-ND vs. Row 5:WT-GAN | 54.38 | -31.95 to 140.7 | No | ns | 0.7532 |
| Row 4:KO-ND vs. Row 5:KO-GAN | 51.1 | -30.29 to 132.5 | No | ns | 0.758 |
| Row 4:WT-GAN vs. Row 4:KO-GAN | -38.18 | -124.5 to 48.15 | No | ns | 0.9886 |
| Row 4:WT-GAN vs. Row 5:WT-ND | 126.3 | 40.00 to 212.7 | Yes | **** | <0.0001 |
| Row 4:WT-GAN vs. Row 5:KO-ND | 127.5 | 41.20 to 213.9 | Yes | **** | <0.0001 |
| Row 4:WT-GAN vs. Row 5:WT-GAN | 106.5 | 36.66 to 176.3 | Yes | **** | <0.0001 |
| Row 4:WT-GAN vs. Row 5:KO-GAN | 103.2 | 16.90 to 189.6 | Yes | ** | 0.0044 |
| Row 4:KO-GAN vs. Row 5:WT-ND | 164.5 | 83.11 to 245.9 | Yes | **** | <0.0001 |
| Row 4:KO-GAN vs. Row 5:KO-ND | 165.7 | 84.31 to 247.1 | Yes | **** | <0.0001 |
| Row 4:KO-GAN vs. Row 5:WT-GAN | 144.7 | 58.35 to 231.0 | Yes | **** | <0.0001 |
| Row 4:KO-GAN vs. Row 5:KO-GAN | 141.4 | 78.93 to 203.9 | Yes | **** | <0.0001 |
| Row 5:WT-ND vs. Row 5:KO-ND | 1.2 | -80.19 to 82.59 | No | ns | >0.9999 |
| Row 5:WT-ND vs. Row 5:WT-GAN | -19.83 | -106.2 to 66.50 | No | ns | >0.9999 |
| Row 5:WT-ND vs. Row 5:KO-GAN | -23.1 | -104.5 to 58.29 | No | ns | >0.9999 |
| Row 5:KO-ND vs. Row 5:WT-GAN | -21.02 | -107.4 to 65.30 | No | ns | >0.9999 |
| Row 5:KO-ND vs. Row 5:KO-GAN | -24.3 | -105.7 to 57.09 | No | ns | >0.9999 |
| Row 5:WT-GAN vs. Row 5:KO-GAN | -3.275 | -89.60 to 83.05 | No | ns | >0.9999 |
